# Novel Virus Discovery and Genome Reconstruction from Field RNA Samples Reveals Highly Divergent Viruses in Dipteran Hosts

**DOI:** 10.1371/journal.pone.0080720

**Published:** 2013-11-18

**Authors:** Shelley Cook, Betty Y.-W. Chung, David Bass, Gregory Moureau, Shuoya Tang, Erica McAlister, C. Lorna Culverwell, Edvard Glücksman, Hui Wang, T. David K. Brown, Ernest A. Gould, Ralph E. Harbach, Xavier de Lamballerie, Andrew E. Firth

**Affiliations:** 1 Department of Life Sciences, Natural History Museum, London, United Kingdom; 2 Department of Plant Sciences, University of Cambridge, Cambridge, United Kingdom; 3 UMR_D 190 "Emergence des Pathologies Virales" (Aix-Marseille Univ. IRD French Institute of Research for Development EHESP French School of Public Health), Marseille, France; 4 Department of General Botany, University Duisburg-Essen, Essen, Germany; 5 Centre for Ecology & Hydrology, Wallingford, Oxfordshire, United Kingdom; 6 Department of Pathology, University of Cambridge, Cambridge, United Kingdom; The Pirbright Institute, United Kingdom

## Abstract

We investigated whether small RNA (sRNA) sequenced from field-collected mosquitoes and chironomids (Diptera) can be used as a proxy signature of viral prevalence within a range of species and viral groups, using sRNAs sequenced from wild-caught specimens, to inform total RNA deep sequencing of samples of particular interest. Using this strategy, we sequenced from adult *Anopheles maculipennis* s.l. mosquitoes the apparently nearly complete genome of one previously undescribed virus related to chronic bee paralysis virus, and, from a pool of *Ochlerotatus caspius* and *Oc. detritus* mosquitoes, a nearly complete entomobirnavirus genome. We also reconstructed long sequences (1503-6557 nt) related to at least nine other viruses. Crucially, several of the sequences detected were reconstructed from host organisms highly divergent from those in which related viruses have been previously isolated or discovered. It is clear that viral transmission and maintenance cycles in nature are likely to be significantly more complex and taxonomically diverse than previously expected.

## Introduction

The emergence of new infectious diseases, many of which are caused by RNA viruses, is a major threat to human, animal and plant health and agriculture. RNA viruses demonstrate remarkable capacity to evolve due to large population size, short generation times and high mutation and recombination rates. Many are also vector-borne, potentially increasing the prevalence and range of a given virus in natural ecosystems. Understanding the mechanisms underlying viral emergence is key for the rational design of antiviral therapies and control strategies. A first step in this process is the characterisation of the true distribution of virus genetic variability, both spatially and taxonomically across different host species. Recent research has demonstrated that there exists a vast diversity of previously undiscovered viruses in the natural environment [1]. For example, a large number of “insect-specific” flaviviruses have been discovered recently in numerous culicine mosquito species (Diptera: Culicidae: Culicinae) and these viral strains are likely to vastly outnumber the pathogenic strains present in the natural environment, including those flaviviruses that cause diseases such as yellow fever and dengue fever in humans [2,3,4,5]. A variety of other novel RNA and DNA viruses have been recently identified in insects [6,7]. Deep sequencing technologies have the potential to provide an unprecedented description of this genetic background, and thus to begin to understand the interactions of pathogenic and “silent” viruses in nature, which may include competitive exclusion, superinfection, recombination and other mechanisms that may be involved in the generation of viral diversity [8,9,30]. 

Invertebrates, including dipterans (order Diptera, the two-winged flies) such as mosquitoes, respond to viral infection via RNA interference (RNAi) or RNA silencing, leading to suppression or elimination of the pathogen via sequence-specific degradation of homologous RNA sequences into small RNAs (sRNAs) of discrete sizes. This has been demonstrated in *Stegomyia aegypti* [=*Aedes aegypti*] mosquitoes infected with dengue virus (DENV) or Sindbis virus (SINV), *Culex quinquefasciatus* mosquitoes orally exposed to West Nile virus (WNV) and *Drosophila* infected with flock house virus (FHV) [10,11,12,13]. Similarly, studies in RNAi-deficient *Anopheles gambiae* mosquitoes showed increased viral dissemination rates and titres of inoculated O’nyong-nyong virus [14]. In this study, we used sRNAs sequenced from mosquitoes and chironomids sampled from the natural environment to inform total RNA deep sequencing of samples of particular interest. Within the mosquitoes, the vast majority of flaviviruses discovered to date have been isolated from culicine mosquito species (subfamily Culicinae, see examples above) as opposed to anopheline mosquitoes (subfamily Anophelinae, for example *An. gambiae*, a major vector for malarial parasites, see example above), despite the fact that species from both groups of Culicidae take bloodmeals. In contrast, the chironomids (Diptera: Chironomidae) are non-biting midges. We planned to sample all three groups in order to test whether related viruses may be discovered in a range of dipterans in the natural environment, potentially related to a shared environment and/or transmission and maintenance cycles aside from blood-feeding.

## Materials and Methods

### Trapping protocol

Approximately 600 mosquitoes and chironomids were collected in June 2011 in the Camargue region of France for screening. Specimens were sampled from a variety of locations using different collection methods to maximise species diversity. Collections were made within and around (i) an ornithological park, (ii) a rice farm and (iii) a rural farmhouse and stables. CDC fan-augmented light traps were supplemented with dry ice for ~8-hour trapping periods between dusk and dawn and placed at various heights above ground. Modified backpack aspirators and hand-held aspirators were used to sample resting specimens throughout the day. Collections of immature stages were made from standing water in the three ecological communities. All necessary authorisations were obtained from respective land owners.

Adult mosquitoes were subdued via refrigeration and sorted according to trap and location under a microscope and over a chill table while still alive, and identified to species using keys to the mosquitoes of Europe [15]. Specimens were then pooled directly into liquid nitrogen. Pools varied from 25-140 individuals. Insect-specific flaviviruses, which have been detected in various locations in southern Europe, have been isolated from culicine, rather than anopheline, mosquitoes in the vast majority of cases and hence one of our sampling and pooling aims was to collect (i) adult anophelines, (ii) adult culicines, (iii) immature anophelines and (iv) immature culicines, from each collection site, plus (v) a pooled sample of chironomids from across all sites, for comparison of viral diversity. 

### Total and small RNA preparation, library construction and Illumina sequencing

RNA was purified from 0.5 g of flies using the Ambion mirVana miRNA Isolation Kit (PE Applied BioSystems, Warrington, England) according to the manufacturer’s instructions for total and small-enriched RNA. Additionally, the latter were DNase-treated. All RNA extractions were stored at -80°C and evaluated on a Bioanalyzer (Agilent Technology, Santa Clara, CA, USA). 

Twelve tagged small-enriched RNA libraries were prepared for single read sequencing using the HiSeq 2000 platform (Illumina, San Diego, CA, USA). Briefly, acrylamide gel purification of RNA bands corresponding to size range ~20–30 nt was conducted. Adaptor sequences were added so that the libraries could be multiplexed, producing 10–19 million reads per tagged library. Based on the sRNA sequencing, we chose three samples for further investigation: sample 1 (140 adult anopheline mosquitoes, female *Anopheles maculipennis* *sensu lato*, from the farm/stables site), sample 7 (adult chironomids pooled across sites) and sample 9 (25 adult culicine mosquitoes, female *Ochlerotatus caspius* and *Oc. detritus* from the rice farm). Since genome assembly from sRNA alone is difficult due to non-uniform and incomplete coverage and short sequence reads, we subjected total RNA samples to HiSeq sequencing in an attempt to assemble full-genome virus sequences.

### Sequence processing, assembly and virus genome identification

Sequence read processing, assembly and virus genome identification were conducted using a custom bioinformatics pipeline. Trimmed sRNA reads were assembled using Velvet [16] and contigs were compared to a database comprising all National Center for Biotechnology Information (NCBI) RNA virus proteins using blastx [17]. For the total RNA samples, raw Illumina reads were trimmed using the FASTX-Toolkit (see hannonlab.cshl.edu/fastx_toolkit), first using fastq_quality_trimmer to trim sequences from the 3' end back to the first position with a quality score greater than or equal to 28, then using fastq_quality_filter to remove any reads with greater than 10% of positions having a quality score ≤ 20, and finally any reads still containing 'N' characters were removed. Of the order of 150–200 million useable reads, with a mean trimmed length of 96 nt per read, were obtained per sample. Reads were then assembled using Velvet [16] with k-mer values ranging from 19 to 29. Contigs were compared to a database comprising all NCBI RNA virus RefSeq proteins using blastx [17,18]. Long contigs (> 600 nt) with, initially, blastx e-values < 0.01 were extracted. Using blastx, these contigs were compared with a cellular proteome database (NCBI, non-redundant protein database), and contigs with better matches to cellular proteins than to RNA virus proteins were removed. The remaining contigs were inspected manually and selected contigs of interest were concatenated with other partially overlapping contigs where possible. These semi-manual assemblies were also compared with automated Oases [19] and PRICE [20] assemblies. Assemblies were tested for possible further 5' and 3' extensions by iteratively searching for reads with 23+ nt perfect terminal overlaps. This enabled the joining of two contigs to form KF298274 (verified by similarity in both contigs to phlebovirus L proteins, and complementary 5' and 3' ends in the assembled KF298274, as expected for members of the family *Bunyaviridae*), and 5' and 3' extensions of KF298265 (verified by Sanger sequencing). Table 1 lists the main contigs of interest. Novel virus-related computationally assembled sequences have been deposited in Genbank (accession numbers KF298264 to KF298277). The raw Illumina reads have been deposited in the NCBI Sequence Read Archive (SRA) under BioProject PRJNA206059.

**Table 1:** Selected contigs.

**contig**	**length (nt)**	**cov^a^**	**CDS(s)**	**SNPs^b^ syn/non**	**closest acc# in NCBI^c^**	**e-value**	**query % coverage**	**max % identity**	**taxonomic identification of closest acc# in NCBI**	**host / source of closest acc# in NCBI**
KF298264^l^	3601	144	11..2518	8/4	EU122229	2 x 10^-137^	97%	34%	Chronic bee paralysis virus	*Apis mellifera* (Insecta)
			1620..3533^d^	15/2	FJ345316	4 x 10^-167^	98%	42%	"	"
			51..1085	0/3	-	-	-	-	-	*-*
KF298265^l^	1958	431	15..1802	11/11	EU122232	5 x 10^-12^	53%	24%	Chronic bee paralysis virus	*Apis mellifera* (Insecta)
			91..609	8/2	FJ345348	6 x 10^-10^	56%	31%	"	"
KF298266	4373	41.1	<2..4336	23/3	AF133431^e^	8 x 10^-48^	60%	24%	St Croix River orbivirus (segment 1)	*Ixodes scapularis* (Arthropoda)
KF298267	6567	12.4	<3..>6566	10/5	JQ308188	0.0	86%	35%	*Culex* flavivirus	mosquito^k^ sp. (Insecta)
KF298268	4531	29.5	413..4471	19/1	GU979419	1 x 10^-10^	29%	23%	*Spissistilus festinus* virus 1	*Spissistilus festinus* (Insecta)
KF298269^l^	1503	98.0	34..1452	0/0	D55668	3 x 10^-41^	77%	30%	Mycovirus FusoV (*Partitiviridae*)	*Fusarium solani* (Fungi)
KF298270^l^	2550	21.9	386..2395	1/0	AB110977	2 x 10^-88^	95%	34%	*Helicobasidium mompa* mitovirus 1-18	*Helicobasidium mompa* (Fungi)
KF298271^l^	3420	320	92..3262	3/4	JX403941	0.0	100%	87%	Mosquito X entomobirnavirus (segment A)	*Anopheles sinensis* (Insecta)
			1891..2694^f^	2/2	"	2 x 10^-102^	100%	63%	"	"
KF298272^l^	3220	207	95..3088	2/2	JX403942	0.0	100%	90%	Mosquito X entomobirnavirus (segment B)	*Anopheles sinensis* (Insecta)
KF298273	4014	27.4	<2..3964^g^	0/1	JQ070376^h^	1 x 10^-46^	65%	25%	Eubenangee orbivirus (segment 1)	mosquito or *Culicoides* sp. (Insecta)
KF298274	7485	17.2	78..7364	33/4	HQ541738^i^	8 x 10^-124^	49%	28%	Gouleako virus (segment L; *Bunyaviridae*)	*Culex* sp. (Insecta)
KF298275	2542	17.8	<2..>2542	1/0	U90136	1 x 10^-21^	64%	26%	*S. cerevisiae* narnavirus 23S RNA	*Saccharomyces cerevisiae* (Fungi)
KF298276^l^	3074	216	<3..3068	120/27	AF039063	1 x 10^-18^	59%	24%	*S. cerevisiae* narnavirus 20S RNA	*Saccharomyces cerevisiae* (Fungi)
KF298277	6557	18.4	742..3378	184/32	-	-	-	-	-	-
			3378..6431^j^	172/40	GU979420	5 x 10^-86^	60%	32%	*Circulifer tenellus* virus 1	*Circulifer tenellus* (Insecta)

^a^ Mean coverage.

^b^ Number of synonymous (syn) and nonsynonymous (non) substitutions among well-supported single-nucleotide polymorphisms.

^c^ Tblastn against non-redundant (nr) database, all taxa.

^d^ Predicted to be expressed as join(11 1618,1620..3533) via +1 ribosomal frameshifting on UUU_C.

^e^ Hit to AB018086 (Chuzan orbivirus segment 1) has a slightly lower e-value (6 x 10^-47^) but greater coverage (83% coverage; 24% identity).

^f^ Predicted to be expressed as join(92 1891,1891..2694) via ‑ 1 ribosomal frameshifting on U_UUU_UUA.

^g^ ORF broken by stop codon at 2408.2410; suggestive of a genome-integrated sequence.

^h^ Hit to AB018086 (Chuzan orbivirus segment 1) has a slightly lower e-value (2 x 10^-43^) but greater coverage (73% coverage; 23% identity).

^i^ Hit to D10759 (Uukuniemi phlebovirus L segment; *Bunyaviridae*) has a slightly lower e-value (4 x 10^-122^) but greater coverage (77% coverage; 24% identity).

^j^ Predicted to be expressed as a fusion with the ORF 742.3378 via ‑ 1 ribosomal frameshifting on G_GAA_AAC.

^k^ Related sequences are generally associated with mosquitoes of the genus *Culex*.

^l^ Verified by Sanger sequencing – GenBank accessions KF298278 to KF298284.

Using tblastn, translated open reading frames (ORFs) in the Table 1 contigs were compared to the NCBI non-redundant nucleotide database, and all showed greatest similarity to RNA virus sequences with e-values < 10^-9^. To test for (known) genome-integrated sequences, contigs were also compared to the NCBI non-redundant nucleotide (all taxa), transcriptome shotgun assembly (all taxa) and whole genome shotgun contigs (Diptera) databases using tblastn and blastn. To test for chimeric sequences (e.g. mis-assembled or transcripts derived from genome-integrated sequences with flanking non-virally derived sequences), regions of contigs that were too divergent to blast-match to known viral sequences were separately compared to the above databases using tblastn and, where relevant, blastn. It should be noted that, due to incompleteness within the public databases, such methods cannot conclusively establish that sequences are not derived from genome-integrated sequences. To test for DNA contamination in the non-DNase treated total RNA samples, reads from the small-RNA-enriched samples were mapped back to the assembled contigs; all 14 contigs were supported by between 19 (KF298270) and ~140,000 (KF298271) reads, and mapped reads were distributed throughout the length of contigs.

**Figure 1:**
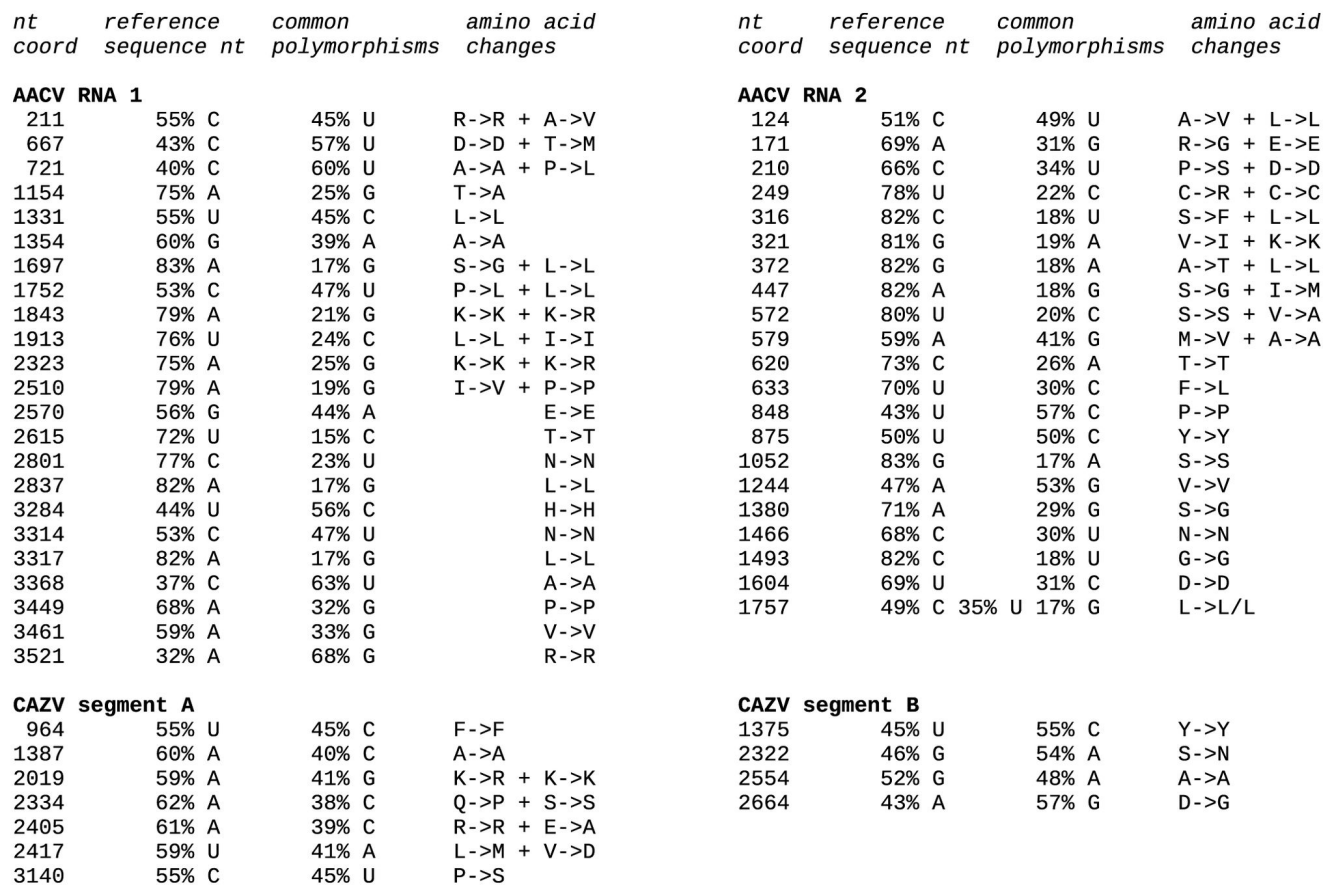
Common nucleotide polymorphisms observed in AACV and CAZV assemblies. Only nucleotide variations with a frequency in the mapped reads of at least 20% of the most common nucleotide present at that position are shown. The nucleotide present in the chosen reference assembly is shown first.

To determine coverage and investigate the identity of single nucleotide polymorphisms, individual reads were mapped back onto assemblies using un-gapped blastn with a wordsize of 10 [17,18]. Reads with full-length matches (or at most 3 nt of terminal mismatch) to virus assemblies, with e-values < 0.001 but with no other restriction on the number of internal mismatches, were mapped to assembly nucleotide coordinates, and the number and identity of nucleotides at each position of each assembly were summed. Single nucleotide polymorphisms with a relative frequency in reads of at least 20% of the most common nucleotide at the position, and an absolute frequency of at least 3 (i.e. present in at least 3 different reads), are shown in Figure 1 for anopheline-associated C virus (AACV) and culicine-associated Z virus (CAZV). Numbers of synonymous and nonsynonymous polymorphisms for all selected contigs are also reported in Table 1.

### Validation and completion of viral genome sequences with Sanger sequencing

To further test for chimeric sequences and other misassemblies, selected contigs (see Table 1) were validated via Sanger sequencing of overlapping RT-PCR products (Genbank accession numbers KF298278 to KF298284). Samples were DNase-treated prior to RT-PCR The PCR products were A-tailed and cloned into the TA vector – pGemT-easy (Promega, Madison U.S.A.), followed by transformation into DH5α cells. Plasmid DNA were extracted by Plasmid DNA miniprep kit (NBS Biologicals Ltd. Cambridge U.K.) and sequenced with both M13F and M13R primers as well as internal primers when necessary (primer sequences available from authors on request).

### Phylogenetic and conservation analyses

Sequences in GenBank related to AACV, CAZV and other selected contigs were identified using tblastn [17,18]. Multiple sequence alignment was performed via MUSCLE [21]. Regions of ambiguous alignment were removed using G-Blocks [22]. Maximum likelihood (ML) phylogenetic trees were estimated via the Bayesian Markov chain Monte Carlo (MCMC) method implemented in MrBayes. All parameters were estimated from the data under default priors and Markov chains were run for a minimum of 20 million generations, with the first 10% of samples discarded as burn-in. Support for nodes was assessed using posterior probability values calculated in MrBayes. All phylogenetic analyses were carried out on the freely available Bioportal server (www.bioportal.uio.no). Due to the highly divergent nature of many of the sequences, alignments were also conducted via COBALT, using conserved domain and local sequence similarity information [23] and neighbour-joining trees constructed via PAUP [24] for comparison. ML trees were all midpoint-rooted for clarity. Due to the high divergence of narnavirus-like sequences, a maximum likelihood tree was not constructed for these sequences. Rather, a simple illustrative neighbour-joining tree was constructed using clustalx [25] with alignment positions containing gap characters excluded. Nodes with < 80% bootstrap support were collapsed.

Conservation at synonymous sites was analysed as described previously [26]. The probability of the conserved absence of stop codons in the entomobirnavirus X ORF occurring by chance (i.e. if the X ORF were actually non-coding) was assessed via pVP2-VP4-VP3-frame alignment codon column shuffling as described previously [27].

## Results

### A new virus, AACV, related to chronic bee paralysis virus

**Figure 2:**
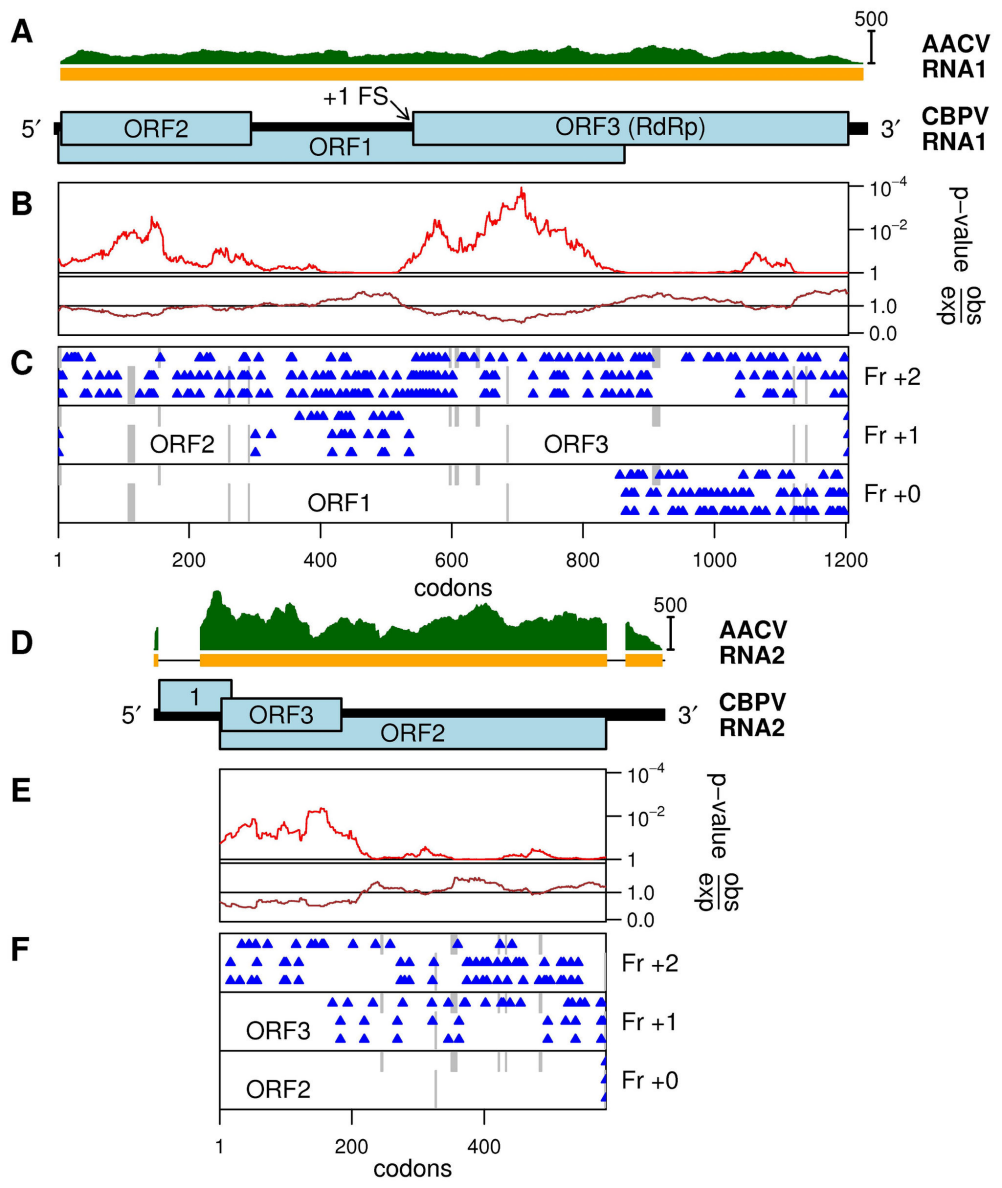
Analysis of CBPV and AACV sequences. (A) Map of the CBPV RNA1 genome segment. The region covered by contig KF298264 (AACV) is indicated by the orange bar. The read coverage density is indicated in green. (B) Analysis of variability at synonymous sites in an alignment of the currently available full-length CBPV sequences (EU122229 and EU122231) and AACV (KF298264). Shown are the degree of variability at synonymous sites in a 75-codon sliding window, relative to the average in the ORF1-ORF3 frameshift fusion (obs/exp), and the corresponding statistical significance (*p*-value). (C) Positions of stop codons in the three forward reading frames in the three sequences (KF298264 - top row of triangles in each panel; CBPV EU122231 and EU122229 - bottom two rows of triangles in each panel). (D, E and F) Corresponding figures for RNA2 (KF298265 - top row of triangles in each panel; CBPV EU122232 and EU122230 - bottom two rows of triangles in each panel). Note that AACV RNA2 lacks a homolog of the CBPV ORF1, and has a shorter 3' UTR than CBPV, as indicated by gaps in the orange bar.

Sequences related to RNA1 and RNA2 of chronic bee paralysis virus (CBPV) were assembled from sample 1 (adult female *Anopheles maculipennis* s.l. mosquitoes from a farm/stables site). CBPV is a positive-sense single-stranded RNA virus with a bipartite genome and no close relatives among sequenced viruses (Figures 2a and 2d) [28,29]. It is a disease agent of adult honey bees (*Apis mellifera*) that causes characteristic “paralysis” symptoms. Recently, the sequences of partly related (but still highly divergent) viruses, Lake Sinai viruses 1 and 2 (LSV1 and 2), were also obtained from *A. mellifera*. The LSVs have around 18% and 25% amino acid identity to CBPV in, respectively, the CBPV RNA1 ORFs 1 and 3, but otherwise they have a rather different genome organization from CBPV [30]. Whereas the LSVs are predicted to encode a tetravirus-like capsid protein in an ORF covering the 3' approximately one-third of their monopartite genome [30], CBPV lacks the 3' ORF on RNA1 and instead one or more capsid proteins are believed to be encoded by RNA2 [28].

**Figure 3:**
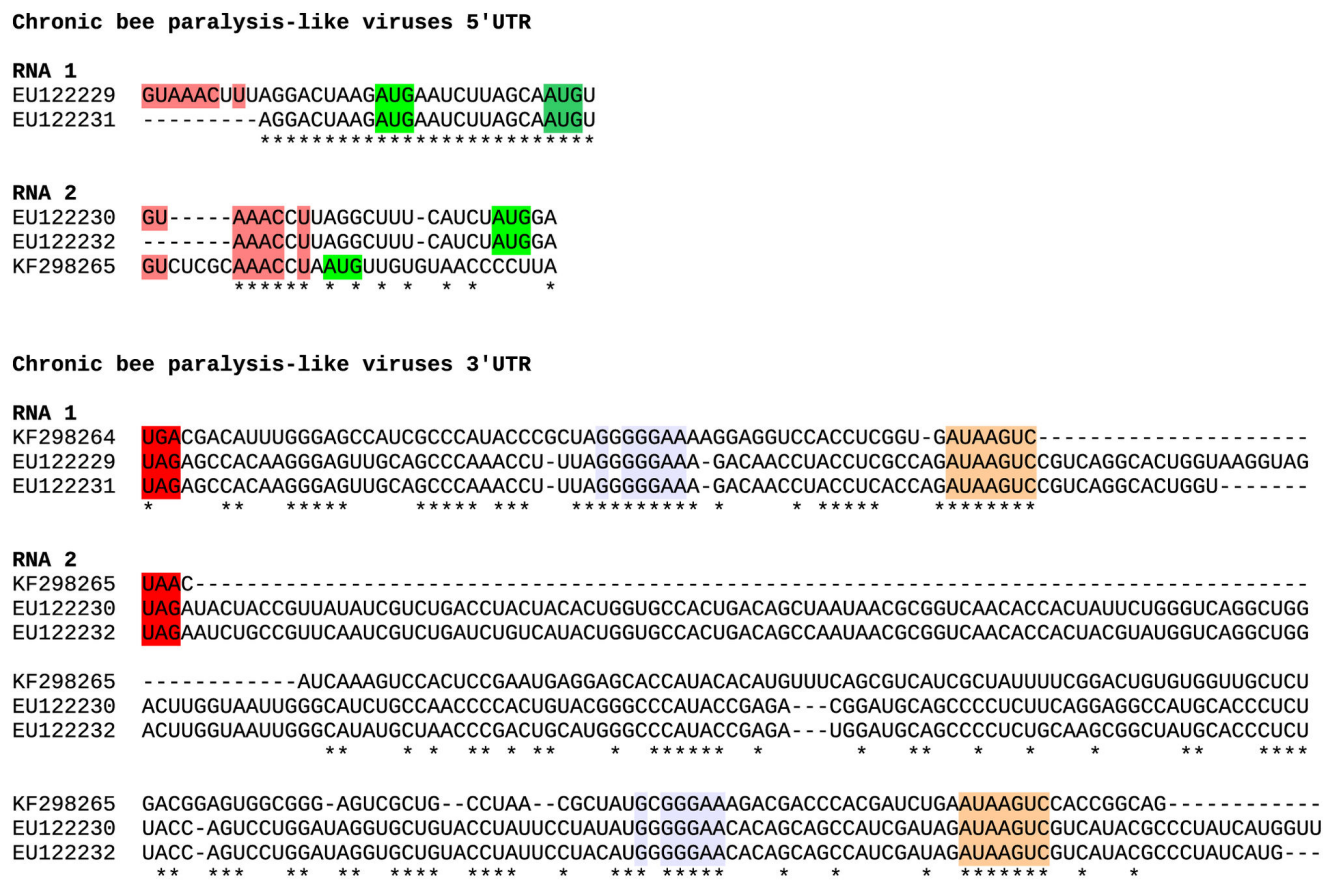
CBPV and AACV 5' and 3' UTR sequences. Initiation and termination codons are highlighted in green and red respectively. CBPV and AACV sequences share common 5' and 3' motifs. GxGGGAA (blue-grey), AUAAGUC (orange) and other motifs are present in the 3' UTR of both RNA1 and RNA2 of both viruses, whereas a GU...AAACxU motif (salmon) is present at the 5' end of CBPV RNA1 and RNA2 and AACV RNA2. The absence of this motif at the 5' end of the AACV RNA1 sequence suggests that this sequence is incomplete (see text).

We were able to assemble apparently nearly complete sequences corresponding to CBPV RNA1 and RNA2 (KF298264, 3601 nt; KF298265, 1958 nt). The new sequences have a CBPV-like rather than LSV-like genome organization and the predicted translation products are similar to those of CBPV, with the exception that RNA2 lacks the CBPV ORF1 (Figure 2d). We call the new (putative) virus anopheline-associated C virus or AACV, where the epithet 'C' reflects the similarity to CBPV. Good coverage was obtained for both RNA1 and RNA2 (mean coverage 144-fold and 431-fold respectively; Figures 2a and 2d; Table 1). The presence of common motifs in the 3' UTRs of CBPV RNAs 1 and 2 that were also present in the 3' ends of AACV RNAs 1 and 2 suggests that the AACV assemblies are nearly complete at their 3' ends (Figure 3). Similarly the presence of common 5' motifs [28] suggests that the AACV RNA2 assembly is nearly complete at the 5' end, whereas the AACV RNA1 assembly is likely to be lacking the 5' end. However, alignment of AACV RNA1 with CBPV RNA1 suggests that only of order 10 codons are missing from the 5' end of ORF1 (not shown).

**Figure 4:**
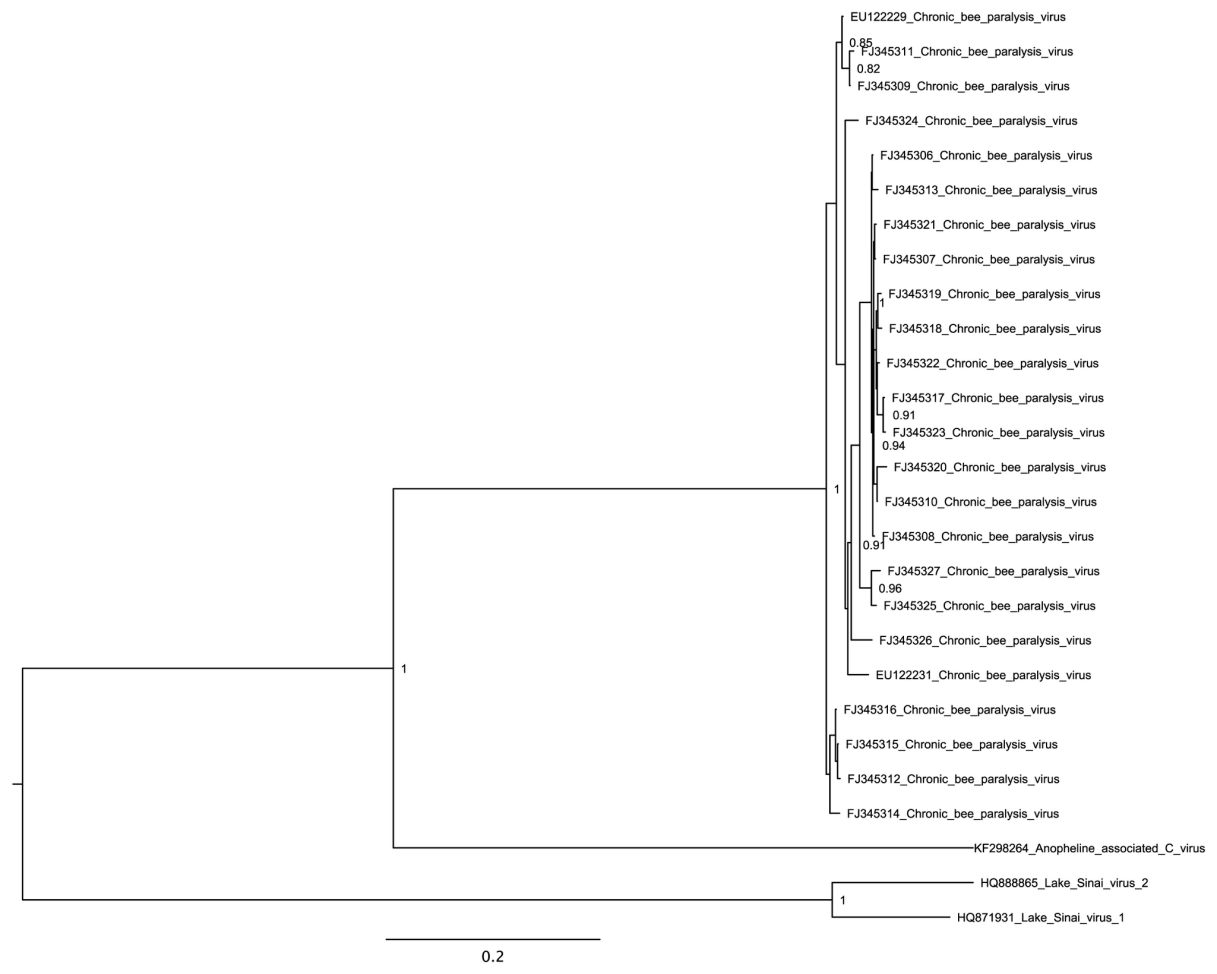
Bayesian maximum likelihood (ML) phylogenetic tree for AACV RdRp and related sequences. The tree is midpoint-rooted and, for clarity, only posterior probability values >80% are shown.

**Figure 5:**
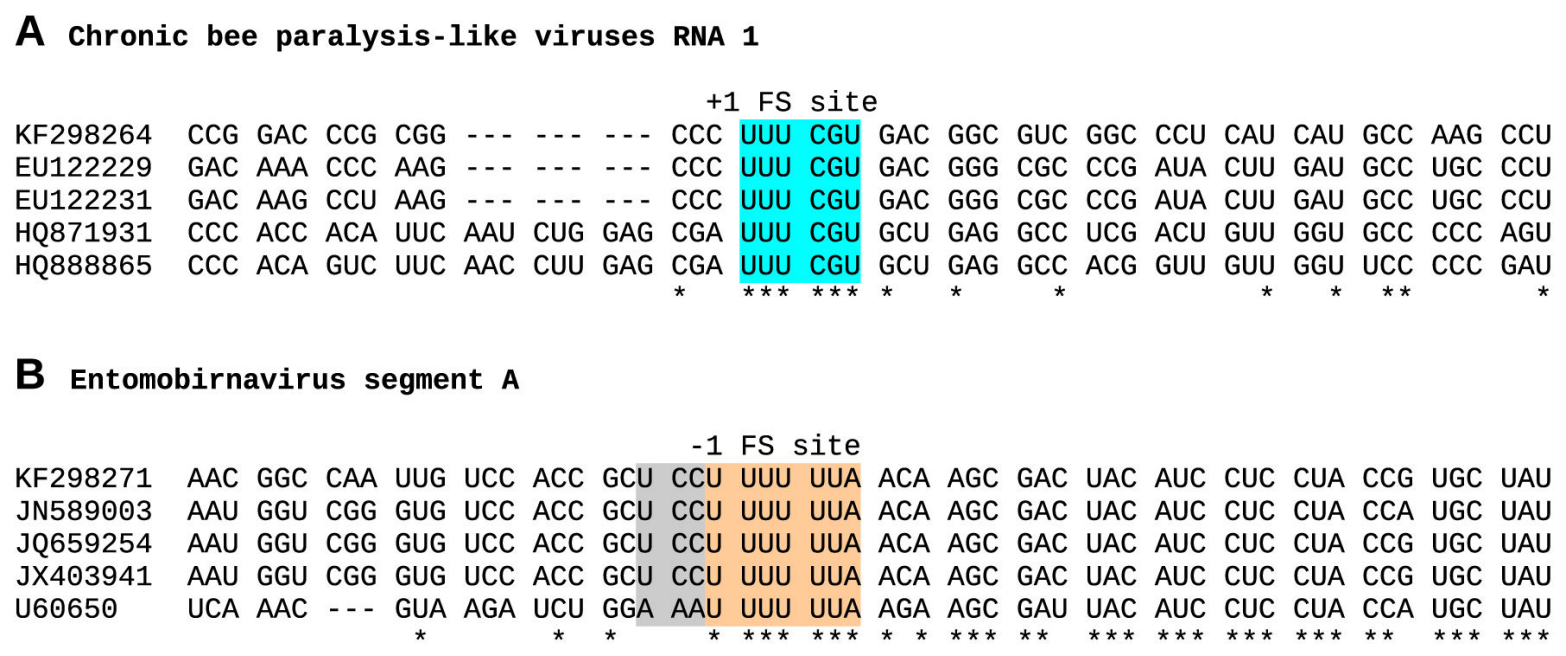
Predicted frameshift sites. (A) In AACV, CBPV and LSVs, +1 frameshifting for RdRp expression is predicted to occur on a conserved UUU_CGU motif (highlighted in cyan; P-site slippage on UUU_C), similar to the site of +1 frameshifting in influenza A virus PA-X expression [32]. KF298264 – AACV; EU122229 and EU122231 – CBPV; HQ871931 – LSV1; HQ888865 – LSV2. (B) In entomobirnaviruses, -1 frameshifting for VP4N-X expression is predicted to occur on a conserved U_UUU_UUA motif (highlighted in orange; tandem P- and A-site slippage), which is a particularly slippery site for -1 frameshifting [38]. This may be further supplemented by some level of tandem P- and A-site -1 slippage on U_CCU_UUU (ESV, CYV, MXV, CAZV) or A_AAU_UUU (DXV) (grey and orange highlighting). Note the increased nucleotide conservation downstream of the U_UUU_UUA motif, consistent with overlapping features. KF298271 – CAZV; JN589003 – ESV; JQ659254 – CYV; JX403941 – MXV; U60650 – DXV.

Nonetheless, the AACV sequences diverge substantially from CBPV, with approximately 33%, 20%, 40%, 16% and 23% mean amino acid identity in, respectively, RNA1 ORFs 1, 2 and 3 and RNA2 ORFs 2 and 3 (CBPV ORF numbering; Figures 2a, 2d and Figure 4). Thus, the addition of these new sequences facilitates comparative genomic analysis of CBPV. The coding status of the overlapping RNA1 ORF2 and RNA2 ORF3 is supported by the conserved absence of stop codons between CBPV and AACV (Figures 2c and 2f) and enhanced conservation at synonymous sites in the overlapping RNA1 ORF1 and RNA2 ORF2 (Figures 2b and 2e). However, it is unlikely that a potential ORF4 previously identified in CBPV RNA2 [28] is utilised since it is not conserved in AACV and, furthermore, is not easily accessible via leaky scanning. This is in contrast to CBPV RNA2 ORFs 1, 2 and 3, which begin, respectively, at the first, second and third AUGs on the message, where the first two AUGs have weak context, thus favouring leaky scanning. The AACV RNA2 appears to lack the CBPV ORF1 (Figure 2d), which may therefore encode an accessory protein that perhaps evolved after the split with AACV (or else was present ancestrally but subsequently lost from AACV). Like CBPV, the AACV RNA1 contains three overlapping ORFs, where ORF3 encodes the RNA dependent RNA polymerase (RdRp) (predicted via hhpred; [31]) and ORF3 is predicted to be translated as a fusion with ORF1 via +1 ribosomal frameshifting at a UUU_CGU motif that is conserved in CBPV, AACV and LSVs 1 and 2 (Figure 5a) [32]. As shown in Figure 4, AACV is more closely-related to CBPV, with AACV+CBPV forming a sister group to LSV1 and 2. The topologies of phylogenetic trees constructed using alignments from either MUSCLE or COBALT were similar.

Single nucleotide polymorphisms (SNPs) were identified by mapping raw trimmed reads onto assembled contigs. Nucleotide variations with a frequency in the mapped reads of at least 20% of the most common nucleotide present at that position are reported in Figure 1. Fifteen out of 17 such SNPs in RNA1 ORF3 are synonymous with respect to the encoded amino acid; this is not surprising as ORF3 encodes the RdRp that is generally highly conserved. The three SNPs detected in RNA1 ORF2 are all non-synonymous with respect to ORF2, but synonymous with respect to the overlapping ORF1, indicating that the product encoded by RNA1 ORF1 is subject to stronger purifying selection than the product encoded by RNA1 ORF2. In RNA2, preference for synonymous substitutions over non-synonymous substitutions occurred in the ORF3 reading frame in the region where ORF3 and ORF2 overlap.

The presence of both virus segments, the high coverage, the presence of common 5' and 3' terminal motifs and the presence of multiple well-supported SNPs that are generally synonymous (except where genes overlap) strongly suggest that the AACV sequences represent a *bona fide* replicating virus. Whereas CBPV and LSVs 1 and 2 infect honey bees, the AACV sequences were obtained from a sample comprising anopheline mosquitoes, and most likely represent a mosquito-infecting virus. Interestingly, all male mosquitoes and females of many species (also sometimes females of some blood-feeders) feed on nectar, in common with honey bees. 

### A new entomobirnavirus, CAZV

A nearly complete entomobirnavirus genome was assembled from sample 9 (adult female *Ochlerotatus caspius* and *Oc. detritus* mosquitoes from a rice field). CAZV sequences were also detected in high prevalence in samples 3 and 10 (female adult culicine mosquitoes and immature culicine mosquitoes from the bird park respectively).

**Figure 6:**
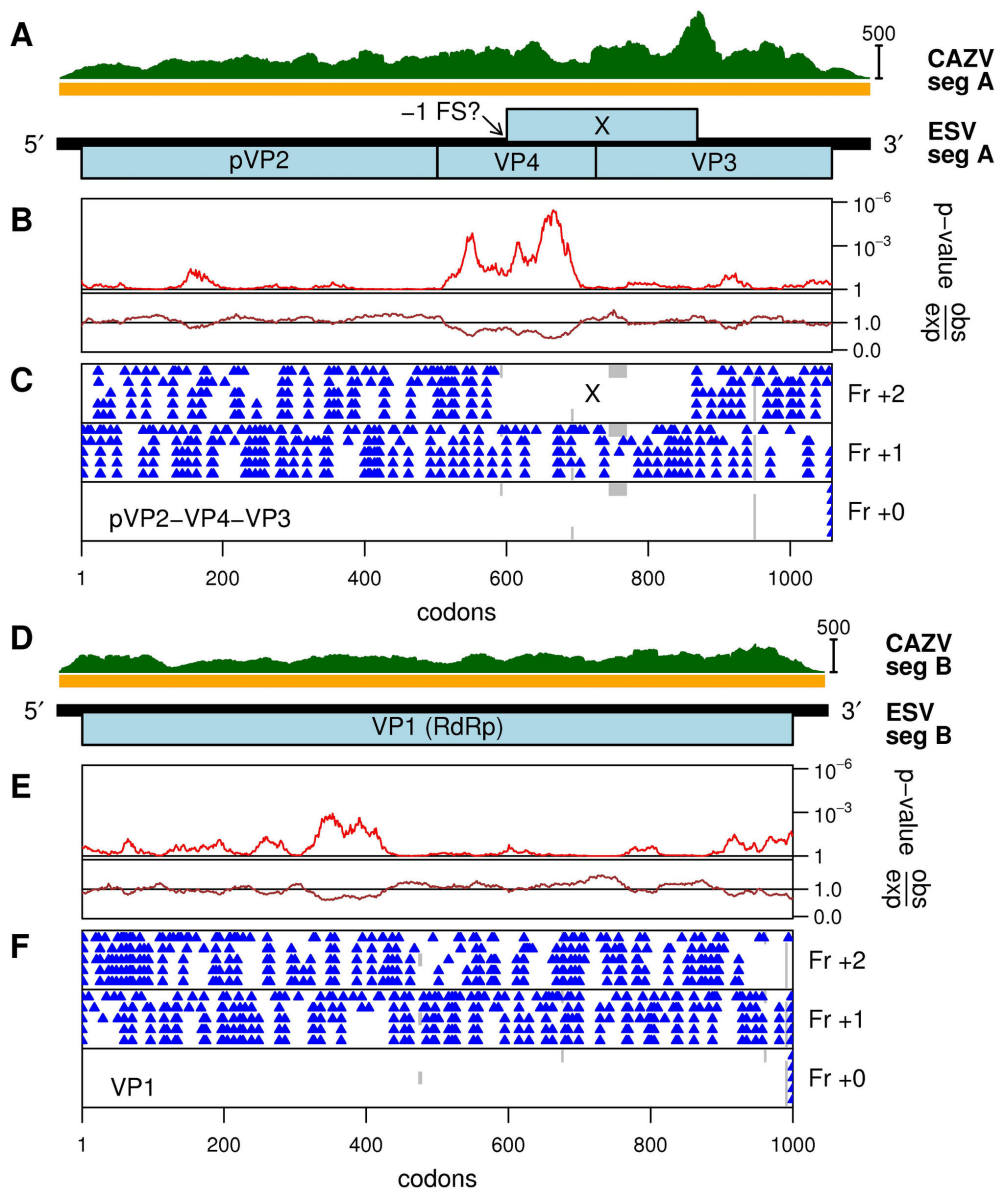
Analysis of entomobirnavirus sequences. (A) Map of ESV genome segment A. The region covered by contig KF298271 (CAZV) is indicated by the orange bar. The read coverage density is indicated in green. (B) Analysis of variability at synonymous sites in an alignment of the currently available entomobirnavirus sequences. Shown are the degree of variability at synonymous sites in a 45-codon sliding window, relative to the average in the pVP2-VP4-VP3 ORF (obs/exp) and the corresponding statistical significance (*p*-value). (C) Positions of stop codons in the three forward reading frames in the five sequences (from top to bottom in each panel: U60650 - DXV, KF298271 - CAZV, JX403941 - MXV, JQ659254 - CYV, JN589003 - ESV). (D, E and F) Corresponding figures for segment B (from top to bottom in each panel: AF196645 - DXV, KF298272 - CAZV, JX403942 - MXV, JQ659255 - CYV, JN589002 - ESV).

**Figure 7:**
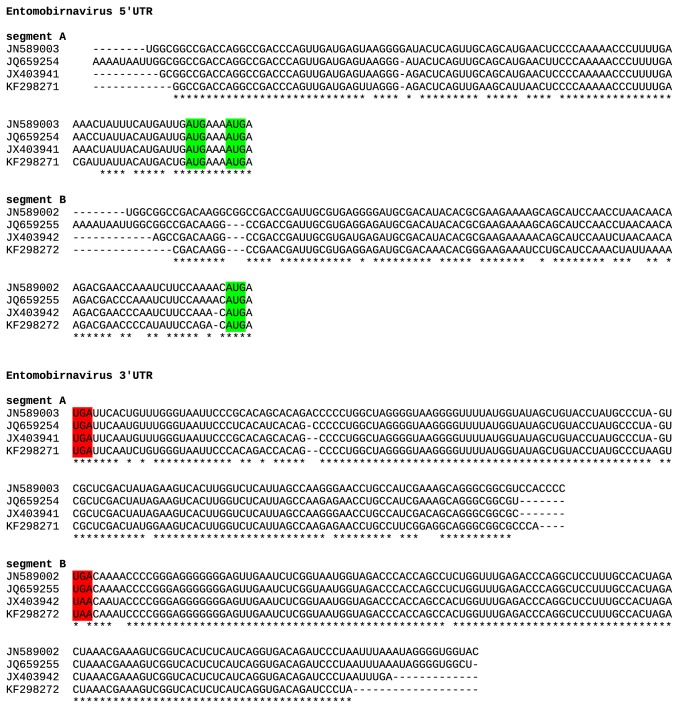
Alignments of entomobirnavirus 5' and 3' UTR sequences, indicating that the CAZV sequences are nearly complete. Initiation and termination codons are highlighted in green and red respectively. Note that both entomobirnavirus segments have upstream AUG codons, although it is not known whether these are utilised. ESV - JN589003/JN589002, CYV - JQ659254/JQ659255, MXV - JX403941/JX403942, CAZV - KF298271/KF298272.

**Figure 8:**
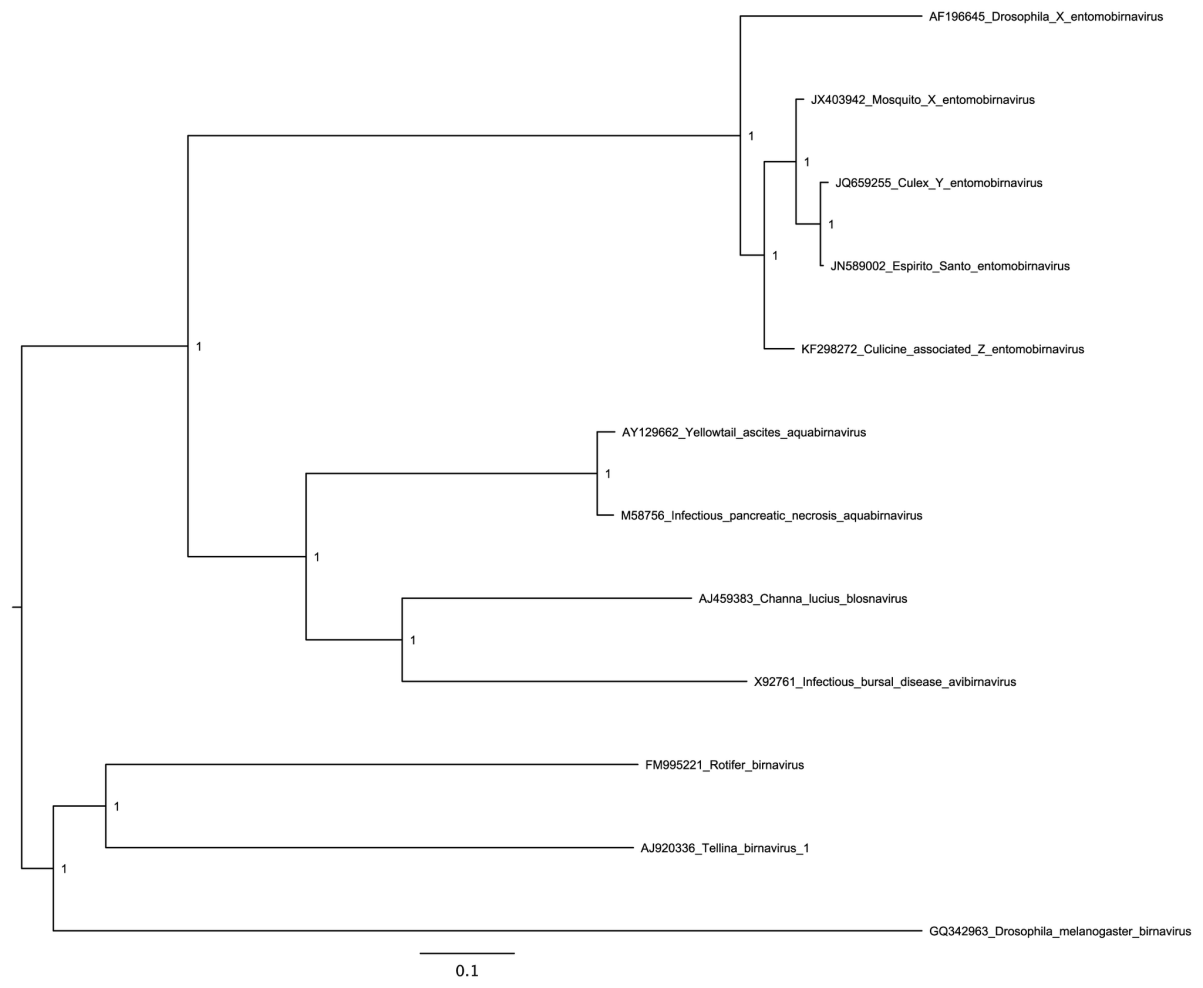
Bayesian maximum likelihood (ML) phylogenetic tree for CAZV RdRp and related sequences. The tree is midpoint-rooted and, for clarity, only posterior probability values >80% are shown.

The genus *Entomobirnavirus* is a member of the family *Birnaviridae*. Members of the family have double-stranded RNA (dsRNA) genomes with two segments, one of which encodes the RdRp (Viral Protein 1, VP1) that is packaged with the viral genomic RNA, whereas the other segment encodes structural proteins pVP2, VP4 and VP3. We designate the new (putative) virus “culicine-associated Z virus” or CAZV. Both segments (A and B) were present, with mean coverage of 320-fold and 207-fold respectively (Figures 6a and 6d; Table 1). Segment A (3420-nt contig) contains a long ORF (1057 codons; predicted to encode pVP2, VP4 and VP3) and a shorter overlapping ORF (X; 268 codons; Figure 6a). Segment B (3220-nt contig) contains a single long ORF (998 codons; predicted to encode the RdRp, VP1; Figure 6d). Comparison of the 5' and 3' ends with those of published entomobirnavirus sequences strongly suggests that both sequences are nearly complete (Figure 7). Currently, only four other full-genome entomobirnavirus sequences are publicly available, for isolates named *Drosophila* X virus (DXV) isolated in the laboratory from *Drosophila melanogaster* cell culture (origin unknown), Espirito Santo virus (ESV) found to replicate in C6/36 cells, *Culex* Y virus (CYV) detected in *Culex pipiens* mosquitoes from Germany, and mosquito X virus (MXV) from *Anopheles sinensis* mosquitoes from China [33,34,35,36,37]. The ESV, CYV and MXV viruses are closely related (>97% amino acid identity) whereas the new virus, CAZV, is more divergent (90% amino acid identity to MXV). However, DXV is even more divergent (67% amino acid identity to MXV). As shown in Figure 8, CAZV appears to fall basal to a clade containing ESV, CYV and MXV, which together form a sister group to the more divergent DXV.

The internal X ORF in segment A is not present in other birnavirus genera so it is of interest to utilise comparative genomic approaches to assess its coding potential. An analysis of nucleotide conservation at synonymous sites in the pVP2-VP4-VP3 ORF of segment A revealed enhanced conservation in the region corresponding to the 5' part of the overlapping X ORF but little evidence for enhanced conservation in the region corresponding to the 3' part of the X ORF (Figure 6a). Nonetheless, a 245- to 268-codon X ORF is conserved in all known entomobirnavirus sequences, which is in itself statistically significant evidence that it encodes a functional product (*p* < 10^-8^). Curiously, enhanced conservation was also observed just upstream of the X ORF. In principle, this could reflect a subgenomic RNA promoter. However, whereas Chung et al., 1996 [33] proposed that the X ORF might be translated from a subgenomic RNA, we favour the alternative proposal of Marklewitz et al., 2012 [36] that the X ORF may be translated via ribosomal -1 frameshifting (as a fusion with the N-terminal half of VP4, giving rise to a VP4N-X protein of around 41 kDa). Evidence for a frameshift expression mechanism includes the presence of a conserved U_UUU_UUA shift site at the 5' end of the X ORF (Figure 5b), and the absence of suitable AUG codons in some entomobirnavirus sequences for independent initiation. U_UUU_UUA is a particularly shift-prone sequence, allowing frameshifting at the level of a few per cent even in the absence of the 3'-proximal RNA structure-based stimulators that are generally required for efficient -1 frameshifting [38,39]. Moreover, all five entomobirnavirus sequences actually have tandem shift-prone heptanucleotides (with U_CCU_UUU or A_AAU_UUU, depending on isolate, overlapping the U_UUU_UUA; Figure 5b), thus allowing for a slightly higher efficiency of frameshifting.

### A flavivirus-like sequence

We identified an incomplete flavivirus-related sequence in sample 7 (adult chironomids pooled across sites) (KF298267; 6567 nt). Flaviviruses have non-segmented single-stranded positive-sense RNA genomes of around 11 kb. The flavivirus genome contains a long ORF that is translated as a polyprotein. Structural proteins are encoded at the 5' end of the genome and non-structural proteins are encoded at the 3' end. In several groups of flaviviruses, additional ORFs are expressed via ribosomal frameshifting [40]. Many flaviviruses infect vertebrates and are transmitted by and replicate within haematophagous arthropods such as mosquitoes and ticks. Some other flaviviruses have no known vector. Still other flaviviruses – the insect-specific flaviviruses (ISFs) – appear to infect only insects [2,3,4,5].

**Figure 9:**
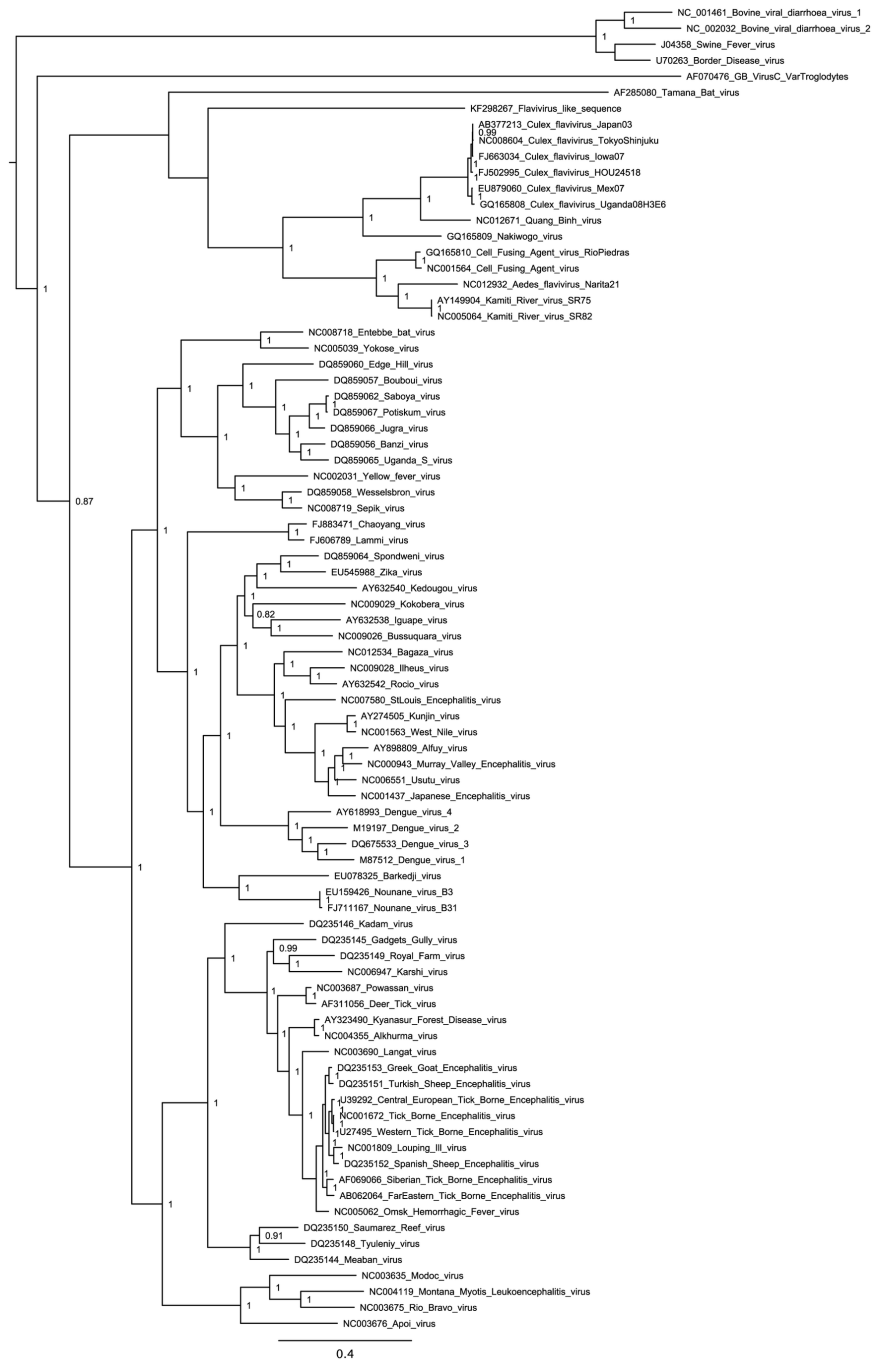
Bayesian maximum likelihood (ML) phylogenetic tree for the flavivirus-like KF298267 and related sequences. The tree is midpoint-rooted and, for clarity, only posterior probability values >80% are shown.

The flavivirus-related fragment, KF298267, contains a single long ORF (<3…>6566) the translation of which showed greatest similarity to *Culex* flavivirus (CxFV; 35% amino acid identity; 86% coverage) and other ISFs. Even so, as shown in Figure 9, KF298267 is potentially divergent enough from the ISFs to be considered a separate subgroup. KF298267 corresponds approximately to the 3' two-thirds of the flavivirus polyprotein ORF. Unlike the rest of the sequence, the approximately 240 amino acids of the N-terminus of the translated ORF, which would correspond to the non-structural protein NS2B and the C-terminal half of NS2A, lack significant similarity (blastp) to CxFV and other flaviviruses. However, the NS2A/2B region is highly variable among flaviviruses (e.g. [41]), and a failure to find significant similarity in the NS2A/2B region is not unexpected between flavivirus clades with similar divergences (i.e. 35% amino acid identity) elsewhere in the polyprotein. Nonetheless, these 240 amino acids contain multiple predicted transmembrane regions, which are a characteristic feature of NS2A/2B. The ISFs, such as CxFV and cell-fusing agent virus, have an overlapping ORF (*fifo*) in the NS2A/2B-encoding region that is translated via ribosomal frameshifting [40]. Since KF298267 does not extend to the 5' end of the NS2A-encoding region, we were unable to test whether KF298267 contains a potential ribosomal frameshift site at this genomic location. However, the 5' end of KF298267 does contain a lengthy overlapping ORF (indeed this is the only other ORF in KF298267 of length >100 codons). This ORF (<2…355) is in the -1 frame relative to the polyprotein ORF, and may represent the 3' end of the *fifo* ORF. 

In sample 7 (adult chironomids pooled across sites), we also detected a sequence (not shown) with similarity to 5' parts of the flavivirus genome; however, the polyprotein ORF was truncated by stop codons. As such, this likely represents a transcribed genome-integrated sequence. In silico, we were unable to link this sequence with KF298267, and were also unable to otherwise extend KF298267 further 5'-ward. Thus, KF298267 may also be derived from a genome-integrated sequence. Indeed, flavivirus-derived integrated sequences have been identified previously in insect genomes [42,43], and a frameshift-interrupted flavivirus-like sequence fragment (KA183058; [44]) exists in a Chironomus riparius transcript shotgun assembly database. Although we identified 15 SNPs in KF298267 (10 synonymous and 5 nonsynonymous), this is not necessarily inconsistent with a genome-integrated sequence, as genome-integrated sequences of viral origin are sometimes subject to exaptation and purifying selection [43,45]. 

### A bunyavirus-like sequence

A sequence related to the large segment that encodes the L protein, or RdRp, of members of the family *Bunyaviridae* was assembled from sample 9 (adult female *Ochlerotatus caspius* and *Oc. detritus* mosquitoes from a rice field) (KF298274; 7485 nt). Bunyaviruses have negative-sense RNA genomes with three segments L (large), M (medium) and S (small). The family includes the genera *Hantavirus*, *Nairovirus*, *Orthobunyavirus*, *Phlebovirus* and *Tospovirus* (reviewed in 46). Hantaviruses generally infect rodents and are aerosol-transmitted. Viruses in the other genera are transmitted by, and also replicate within, arthropod vectors such as mosquitoes, ticks and phlebotomine flies. Trans-ovarial transmission has been reported in some arthropods. Nairoviruses, orthobunyaviruses and phleboviruses infect various vertebrates. Tospoviruses infect plants and are transmitted by thrips. KF298274 contains a single long ORF of 2429 codons that shows greatest similarity to members of the genus *Phlebovirus*, and the currently unclassified Gouléako virus (GOUV). GOUV, which has been detected in mosquitoes of the genera *Anopheles*, *Culex* and *Uranotaenia*, may be insect-specific since it replicates in mosquito cells but not in various vertebrate cell lines that have been tested [47]. As with other members of the *Bunyaviridae*, we found that the 5' and 3' termini of KF298274 were complementary (5'-GCAAAACA ... UGUUUUGC-3'), but different from the 5' and 3' termini of phleboviruses and GOUV. The 5'-3' complementarity suggests that our assembly represents a complete genome segment. We were not however able to detect the M and S segments, though this could simply be because they were two divergent from known sequences to be recognized. It should be noted that bunyavirus mRNAs typically contain 10-18 non-templated nucleotides at the 5' end derived, along with the 5' cap, from the 5' termini of host mRNAs. Such chimeric reads were not observed. However, for viruses with capped mRNAs, coverage of the 5' end is likely to come largely from the negative strand (or uncapped forms of the positive strand) since the 5' cap interferes with ligation of the HiSeq adaptors.

**Figure 10:**
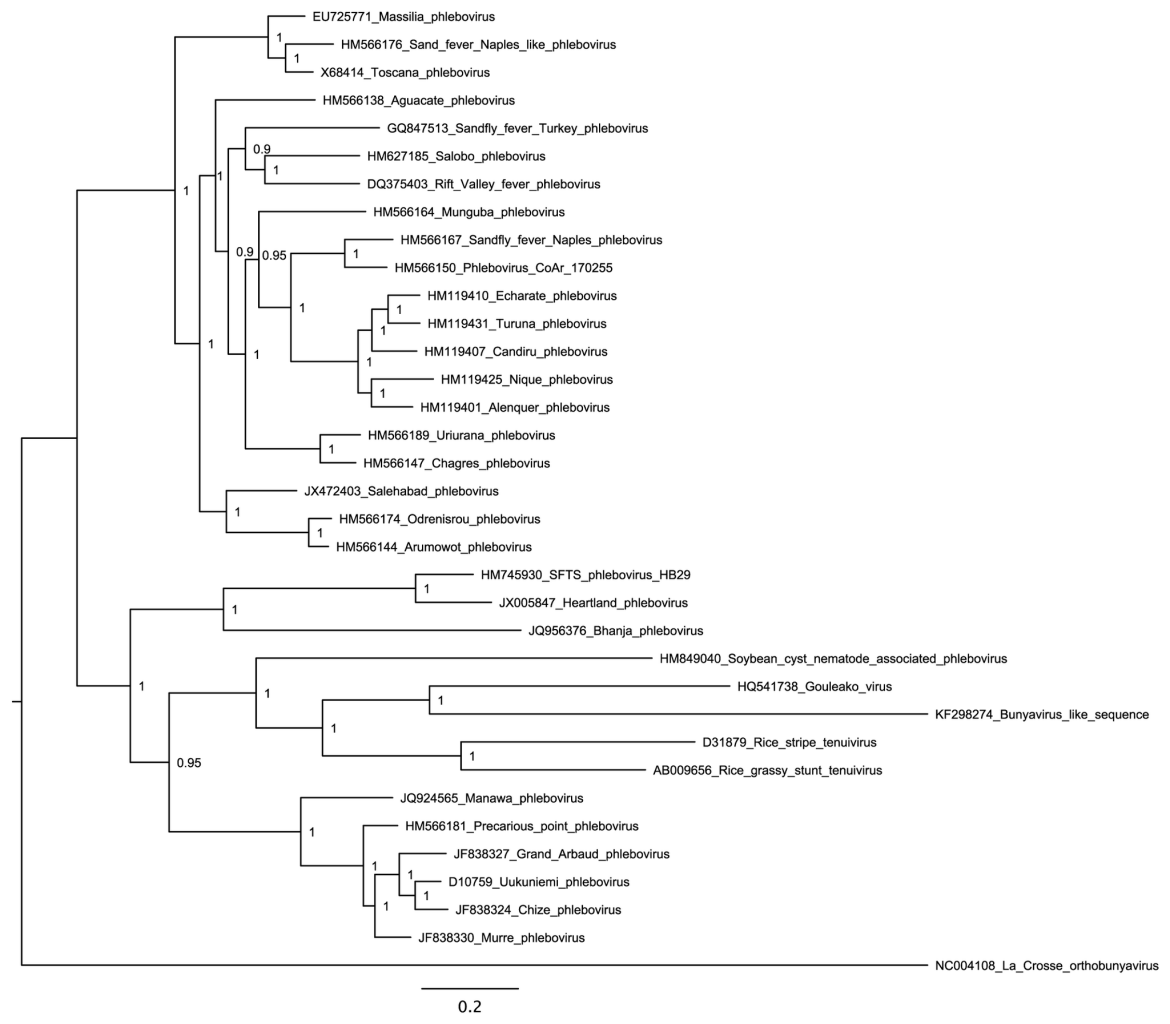
Bayesian maximum likelihood (ML) phylogenetic tree for the bunyavirus-like KF298274 and related sequences. The tree is midpoint-rooted and, for clarity, only posterior probability values >80% are shown.

Although related, KF298274 is nonetheless highly divergent from phleboviruses and GOUV (Table 1; Figure 10). This, and the different terminal sequences, which are normally largely conserved within the genera of the *Bunyaviridae*, suggests that KF298274 represents a new genus. Although phlebovirus-like sequences have been found integrated into arthropod genomes [43], the presence of putative terminal motifs argues against KF298274 being derived from a genome integrant since, when transcribed, genome-integrated sequences are unlikely to utilise the original virus transcript termini. By mapping raw trimmed reads to the assembled sequence, we identified 37 SNPs in the L ORF, 33 of which were synonymous.

### Orbivirus-like sequences

**Figure 11:**
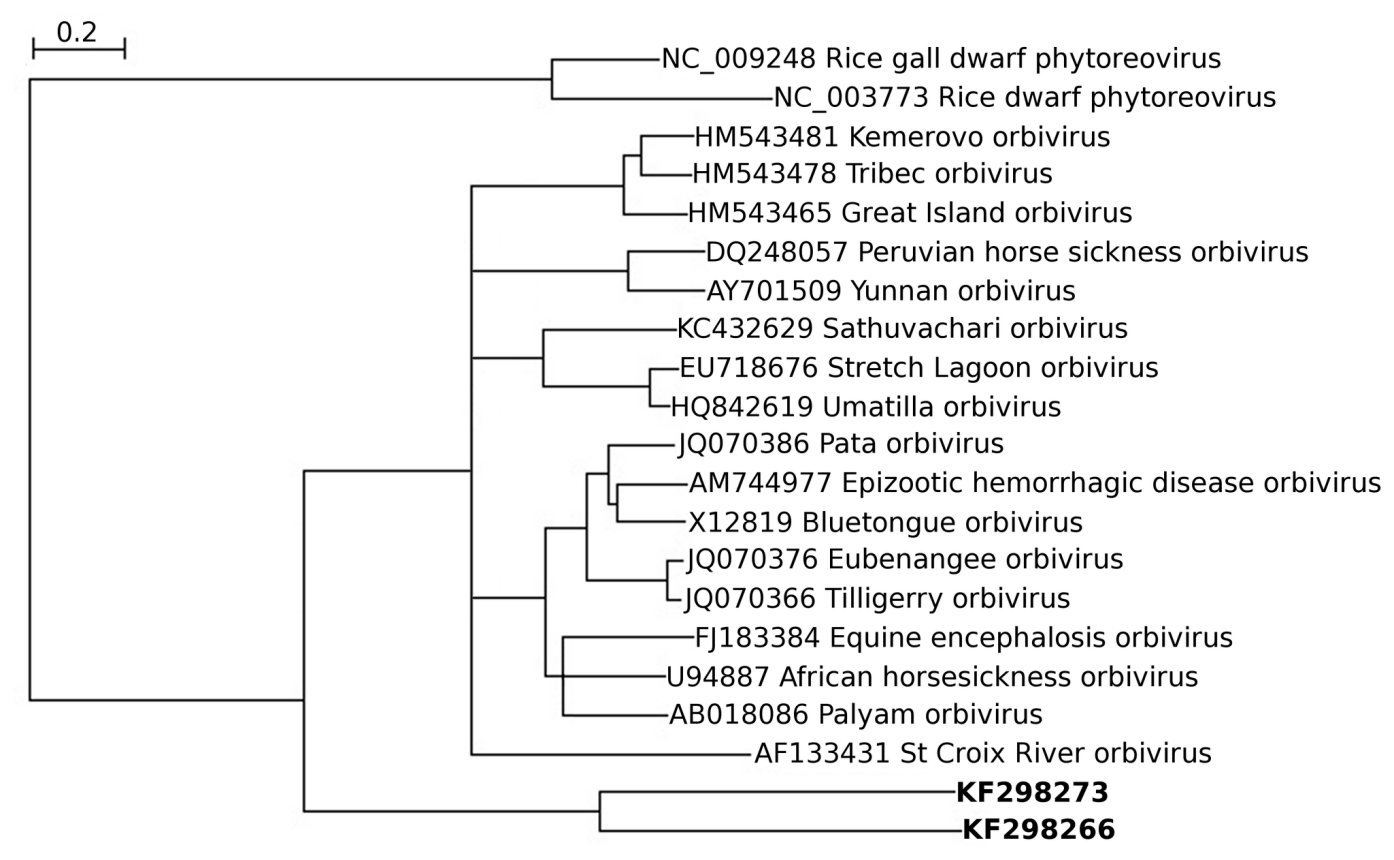
Neighbour-joining (NJ) phylogenetic tree for the orbivirus-like KF298266 and KF298273 and related sequences. Nodes with <80% bootstrap support have been collapsed.

Orbiviruses form a genus in the family *Reoviridae*. Members of the family have a segmented dsRNA genome, with typically 9-12 segments (10 in the case of orbiviruses; [48,49]). Orbiviruses infect vertebrates and are transmitted by, and also replicate in, arthropods such as gnats, mosquitoes, phlebotomine flies and ticks. We detected sequences in samples 1 and 9 (adult anopheline mosquitoes from a farm/stables site and adult culicine mosquitoes from a rice field respectively) with similarity to the orbivirus VP1/RdRp-encoding segment (segment 1). We were, however, unable to extract other segments, although it is not clear whether this was because the other segments were absent (e.g. if the detected sequences are derived from genome-integrated sequences) or because the other segments are too divergent from sequenced orbiviruses to reliably identify via our blast analysis. In any case, these sequences are highly divergent from known orbivirus sequences (Figure 11; Table 1), though orbivirus RdRps are more closely related to those putatively encoded by KF298266 and KF298273 than they are to the RdRps of other reoviruses. The orbivirus-like sequence detected in sample 9 (25 adult female *Ochlerotatus caspius* and *Oc. detritus* mosquitoes from a rice field) (KF298273; 4014 nt) contained an internal stop codon within the VP1 ORF, and just one (nonsynonymous) SNP, consistent with a transcribed genome-integrated sequence. Curiously, the single SNP occurred at the internal stop codon (UAG to CAG in 14 of 43 reads). On the other hand, 23 synonymous and just 3 nonsynonymous SNPs were detected within the VP1 ORF of the orbivirus-like sequence detected in sample 1 (140 adult female *Anopheles maculipennis* s.l. mosquitoes from a farm/stables site) (KF298266; 4373 nt), which may be suggestive of a *bona fide* replicating virus. Related sequence fragments are present in *Anopheles funestus* transcriptome shotgun assembly databases [50], notably the non-overlapping sequences EZ920122 (269 nt; 85% amino acid identity to KF298266) and EZ922704 (207 nt; 68% amino acid identity to KF298266). It is not clear whether such sequences derive from cellular transcripts or from infecting viruses. Although orbivirus segments have conserved (but not fully complementary) 5'- and 3'-terminal sequences of around 6 nt, the motifs differ slightly between species, and considerably between different genera within the family *Reoviridae*. The 5' and 3' termini of KF298266 and KF298273 are not obviously similar to orbivirus conserved motifs, so it is not clear whether or not these sequences represent complete viral segments.

### Sequences related to *Spissistilus festinus* virus 1 and *Circulifer tenellus* virus 1

From samples 7 (chironomids) and 9 (culicine mosquitoes), we identified sequences related to *Spissistilus festinus* virus 1 (SpFV1) and *Circulifer tenellus* virus 1 (CiTV1) [51]. SpFV1 and CiTV1, which were isolated from the hemipteran insects *S. festinus* and *C. tenellus*, respectively, are dsRNA viruses with monopartite genomes of around 8 kb. SpFV1 and CiTV1 are not known to be encapsidated or to encode a capsid protein [51]. Their genomes contain two long terminally overlapping ORFs, where the 3' ORF encodes the RdRp and is believed to be translated as a fusion with the 5' ORF via -1 ribosomal frameshifting at a G_GGA_AAC motif in SpFV1 and a G_GUA_AAC motif in CiTV1 [51]. The 5' ORF encodes a protein of unknown function. Both SpFV1 and CiTV1 have long 5' UTRs containing many AUG codons, and so translation of ORF1 is likely to be IRES- or shunting-dependent.

From sample 7 (adult chironomids pooled across sites), we assembled a 4531-nt contig (KF298268) that contained a long ORF (413.4471) with limited but nonetheless significant amino acid identity to SpFV1 ORF1 (Table 1) and also CiTV1 ORF1. The long 5' UTR is consistent with SpFV1 and CiTV1. Notably, the sequence contains a potential slippery site for -1 frameshifting 5'-adjacent to the ORF1 stop codon (G_GAA_AAC_uaa; ORF1 termination codon in lower case; [39]), and the -1 frame ORF (ORF2) continues to the end of the contig. However, we were unable to assemble the bulk of ORF2 from sample 7. Within ORF1, we detected a number of SNPs, of which 19 of 20 were synonymous. From sample 9 (female adult culicine mosquitoes, specifically *Ochlerotatus caspius* and *Oc. detritus* from a rice field), we assembled a 6557-nt contig (KF298277) that displayed similarity to CiTV1 and SpFV1 in ORF2. This contig apparently contained complete ORF1 (742…3378) and ORF2 (3378…6431) sequences, a long 5' UTR and at least some 3' UTR. Once again, the ORF1 stop codon was preceded by a potential slippery site for ‑1 frameshifting (G_GAA_AAC_uga; ORF1 termination codon in lower case). A large number of SNPs were identified in both ORFs (184 synonymous, 32 nonsynonymous in ORF1; 172 synonymous, 40 nonsynonymous in ORF2).

Although highly divergent (around 42% amino acid identity), SpFV1 and CiTV1 share regions of considerable nucleotide identity at the 5' and 3' termini [51]. Similar sequences were not found in KF298277, although KF298277 is considerably more divergent; thus, it is not clear whether KF298277 represents a complete genome sequence. Blast analysis of KF298277 revealed related sequence fragments in AAGE02000678, a *Stegomyia aegypti* whole genome shotgun sequence. Here, two adjacent ORF2-related fragments of ~250 and ~210 codons are present in AAGE02000678, separated by an unrelated insert of ~5500 nt that includes sequences similar to retrotransposons. Thus (bar misassembly) genome integration of related viruses appears to occur. A number of related viral sequences were also identified in searches of the NCBI transcriptome shotgun assembly database, including JP306102 from *Lepeophtheirus salmonis* (salmon louse, an arthropod), JP089782 from *Mengenilla moldrzyki* (a strepsipteran insect), and EZ959541, HP663044, EZ941741, EZ958863 and HP643964 from *Bemisia tabaci* (silverleaf whitefly, an hemipteran insect). Potentially, these sequence fragments may derive from either transcribed genome-integrated sequences or from infecting viruses. Multiple sequence alignment via both MUSCLE and COBALT produced data sets with numerous highly variable regions; neither manual trimming nor GBlocks stripping of regions of ambiguous alignment produced satisfactory results. Hence, phylogenetic trees were not estimated for the sequences related to SpFV1 and CiTV1.

### Narnavirus-like sequences

In sample 9 (adult female *Ochlerotatus caspius* and *Oc. detritus* mosquitoes from a rice field), we identified two sequences related to the *Saccharomyces cerevisiae* 20S and 23S RNA narnaviruses [52]. The genera *Narnavirus* and *Mitovirus* comprise the family *Narnaviridae*. An additional member of the family – *Phytophthora infestans* RNA virus 4 – was recently reported [53]. Members have a monopartite single-stranded positive-sense RNA genome that typically contains a single ORF that encodes the RdRp. Curiously, the narnavirus RdRp appears to be more closely related to the RdRp of members of the genus *Ourmiavirus* than to the mitovirus RdRp. However ourmiaviruses (which infect plants) are not grouped with the *Narnaviridae* because they have tripartite genomes, where the other two genome segments are believed to have a separate origin via inter-genus/family reassortment [54].

Contigs KF298275 (2507 nt) and KF298276 (3074 nt), which have about 40% amino acid identity, were each spanned (up to 835 and 1022 codons respectively) by an open reading frame, the translation of which bears similarity to the RdRp of *Saccharomyces cerevisiae* 20S and 23S RNA narnaviruses. The sequences are nonetheless highly divergent from *Saccharomyces cerevisiae* 20S and 23S RNA narnaviruses, with amino acid identities, averaged over the whole ORF, of just 12–15%. By searching the NCBI Transcriptome Shotgun Assembly (TSA) database, we identified six other sequences (lengths 2159 to 3097 nt) related to KF298275 and KF298276, namely GACI01002802 (from *Uromyces appendiculatus*), GACM01002912 (from *Phakopsora pachyrhizi*), and GAIR01011807, GAIR01012025, GAIR01012062 and GAIS01005902 (all from *Puccinia striiformis*). These three species are Basidiomycota fungi and it may be conjectured that the six sequences derive from narna-like viruses infecting these hosts. Thus it seems likely that KF298275 and KF298276 derive from fungal or possibly protist contaminants of the insect preparations, either external or internal (e.g. gut contents).

KF298275 had a mean coverage of 17.8-fold and only one SNP was identified. In contrast, KF298276 had a mean coverage of 216-fold and 147 SNPs were identified. These were predominantly synonymous (120 SNPs) with respect to the RdRp amino acid sequence. The large number of SNPs suggests that KF298276 derives from a *bona fide* virus rather than a transcribed genome-integrated sequence. The *Saccharomyces cerevisiae* 20S and 23S RNA narnaviruses have complementary terminal motifs 5'-GGGGC...GCCCC-3', and short UTRs (6-12 nt 5' and 12-59 nt 3') [52]. Complementary terminal motifs were not, however, found in KF298275 or KF298276, and this, together with the absence of a 5'-proximal AUG codon in the RdRp ORF, suggests that even KF298276 may not represent a complete genome.

**Figure 12:**
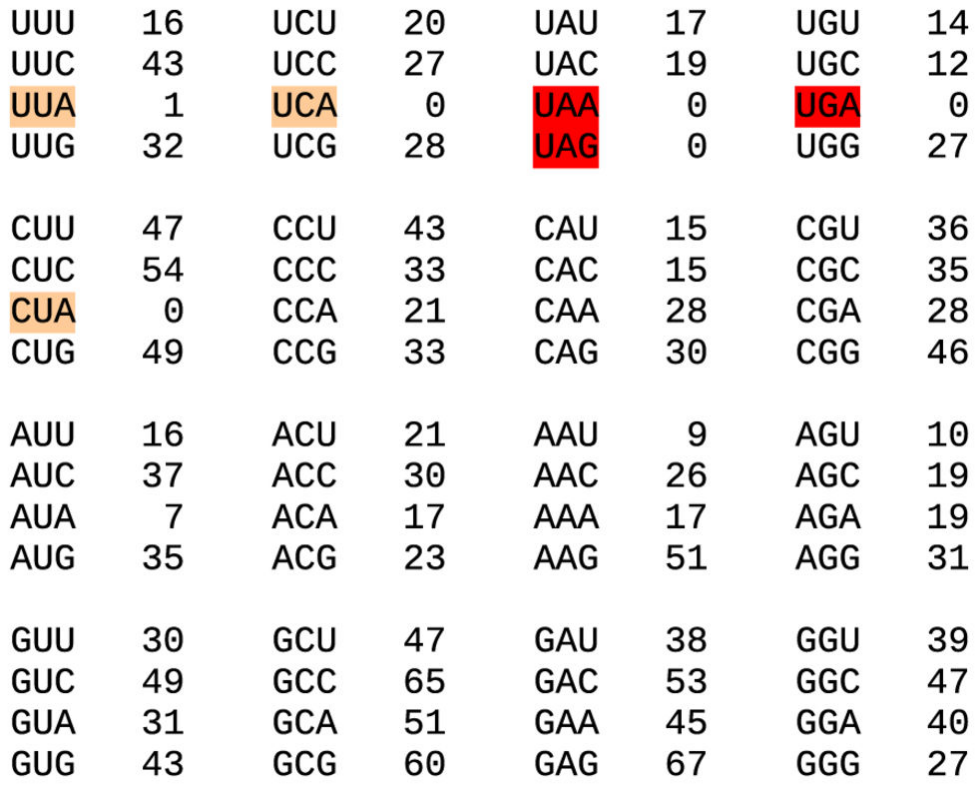
Codon usage statistics for the RdRp ORFs in the narnavirus-like contigs KF298275 and KF298276. In-frame forward read-direction stop codons (red) are necessarily absent. Reverse complements of in-frame but reverse read-direction stop codons are highlighted in orange; a single UUA codon corresponds to the UAA stop codon of the >1000-codon reverse-read-direction ORF in KF298276.

Unusually, both contigs were also spanned (up to 835 codons and up to 1009 codons respectively) by another open reading frame in the reverse read direction. In general, an open reading frame of such a length would not be expected to occur by chance provided that the nucleotide composition is not highly biased (here it is 20% A, 28% C, 31% G, 21% U). On the other hand, despite the production of a negative sense copy of the genome during replication, reverse read-direction ORFs are not expected to be expressed in positive-sense RNA viruses. Moreover, most of the 147 SNPs were nonsynonymous with respect to the reverse read-direction ORF, although they never resulted in the introduction of a stop codon. In both contigs, the reverse direction ORF is in-frame with the forward direction RdRp ORF; thus, one possible explanation for the reverse direction ORF is simply that selection in the RdRp ORF acts strongly against usage of the codons UUA, UCA and CUA, i.e. the reverse complements of the stop codons UAA, UGA and UAG. This might occur if the virus infects an organism in which these codons are rarely or never used, e.g. due to limiting or absent cognate tRNAs. However we are not aware of any eukaryotic organism for which this is the case. Moreover, RdRp codon usage tables for these contigs do not reveal any other codons that are so rarely used (Figure 12). Thus the coding status of the very long reverse direction ORF in each of these contigs remains intriguingly mysterious.

**Figure 13:**
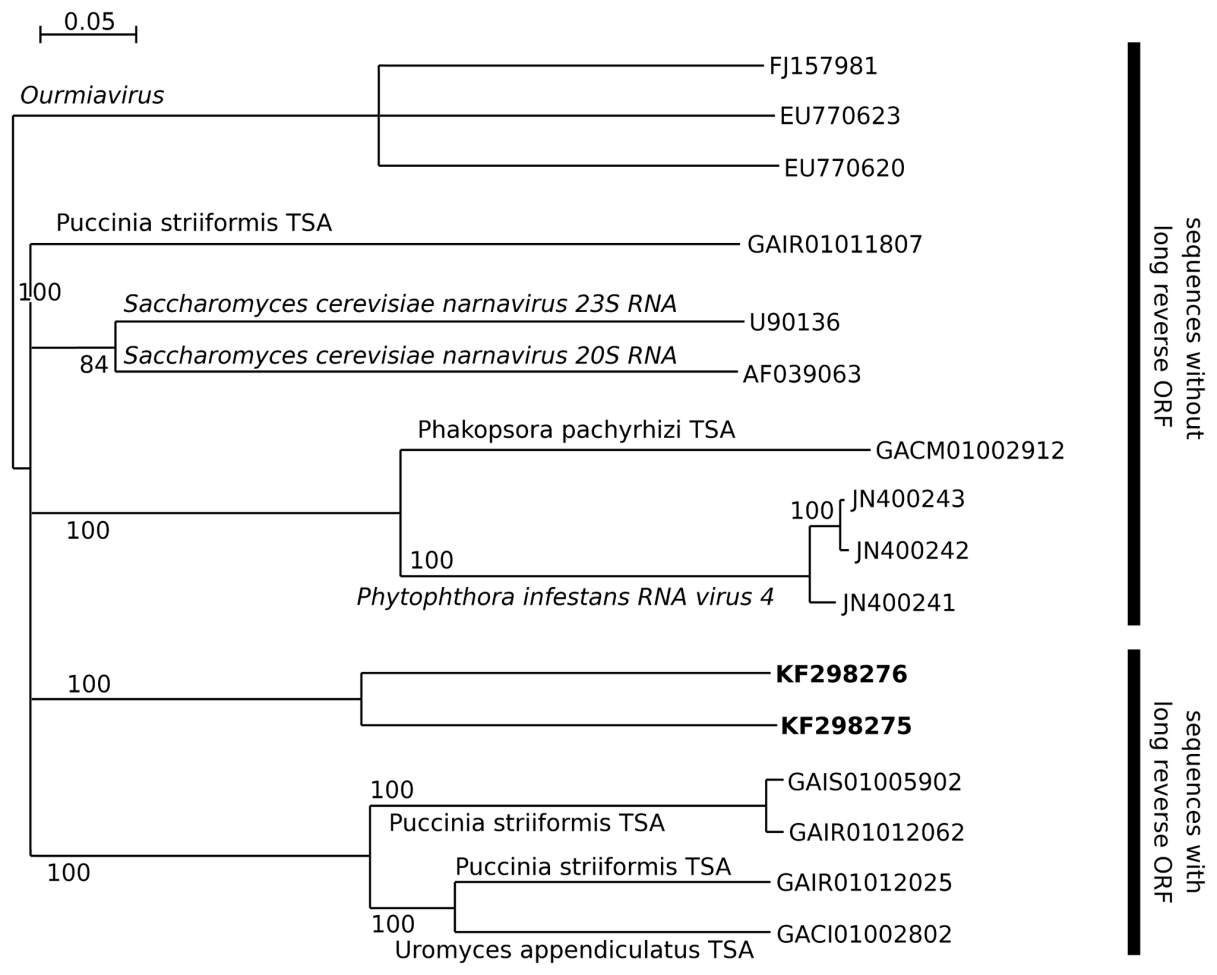
Neighbour-joining phylogenetic tree for the RdRp amino acid sequences of the narnavirus-like KF298275 and KF298276 and related sequences. Nodes with <80% bootstrap support have been collapsed.

A long reverse-direction ORF is not present in the *Saccharomyces cerevisiae* 20S and 23S RNA narnaviruses. However, four of the six TSA sequences, namely GACI01002802 (from *Uromyces appendiculatus*) and GAIR01012025, GAIR01012062 and GAIS01005902 (from *Puccinia striiformis*) do have a long reverse-direction ORF overlapping all or nearly all of the RdRp ORF. These four sequences form a phylogenetic clade distinct from the sequences without the reverse-direction ORF (Figure 13). In these cases, the host species may be assumed to be the TSA target and these hosts appeared to use the UUA, UCA and CUA codons normally in other transcripts, thus suggesting that the long reverse-frame ORF is not an artefact of codon usage in the RdRp ORF. This, together with the extreme divergence between these sequences (e.g. 53% amino acid identity between GAIR01012025 and GAIR01012062, and only around 22% amino acid identity between GAIR01012062 and KF298276, in the RdRp sequence), suggests that the reverse-direction ORF may represent a *bona fide* protein-coding sequence. 

### Sequences similar to other RNA viruses of fungi and plants

Mitoviruses, also in the family *Narnaviridae*, infect the mitochondria of fungi and as a result they use the mitochondrial genetic code where UGA encodes tryptophan instead of signalling translation termination. We detected several distinct mitovirus-like sequences in sample 7 (adult chironomids pooled across sites), one of which, KF298270 (2550 nt), is listed in Table 1. This sequence potentially represents a full- or near full-length virus genome and has a lengthy 5' UTR and complementary terminal sequences (5'-gcaagAGGACACACGC...GCGUGUGUCCU-3'; complementary nucleotides in capitals), as has been observed previously for some other mitoviruses [55]. However, only one SNP was detected for this contig.

Partitiviruses (family *Partitiviridae*) infect fungi and plants. They have bipartite dsRNA genomes with one segment encoding the RdRp and the other segment encoding a capsid protein [56]. We detected partitivirus-like sequences in samples 7 and 9, one of which, KF298269 (1503 nt), is listed in Table 1. This sequence potentially represents a full- or near full-length RdRp-encoding segment. However, despite relatively high coverage (98-fold), no SNPs were detected for this contig. Blast analysis revealed many related sequences in the NCBI transcriptome shotgun assembly and whole genome shotgun sequence databases. Some of the former may derive from infecting viruses. Nonetheless, partitivirus sequences are commonly integrated into the genomes of cellular organisms including arthropods [57]. KF298269 had notably higher similarity (87% coverage, 42% identity) to a region of at least one of the whole genome shotgun sequences (AAPT01020539; *Drosophila grimshawi*; see also 57) than to the most closely related virus sequence in GenBank (Table 1). Other highly significant matches (68-69% coverage, 31-34% identity) were found in the dipterans *Lutzomyia longipalpis* (AJWK01014410) and *Phlebotomus papatasi* (family Psychodidae, phlebotomine sand flies) (AJVK01006534). Nonetheless, these sequences are still highly divergent from KF298269 and we are unable to determine whether KF298269 derives from a *bona fide* partitivirus or from a transcribed genome-integrated partitivirus. Multiple sequence alignment via both MUSCLE and COBALT produced data sets with numerous highly variable regions, and neither manual trimming nor GBlocks stripping of regions of ambiguous alignment produced satisfactory results. Hence, phylogenetic trees were not estimated for the mitovirus-like or partitivirus-like sequences.

## Discussion

We detected and obtained nearly full-genome sequence for a potential new virus, AACV, related to chronic bee paralysis virus (CBPV). AACV sequences were assembled from adult anopheline mosquitoes, whereas CBPV was isolated from the honey bee, *Apis mellifera*. To our knowledge, these are the first reported sequences of a dipteran-infecting CBPV-like virus. A nearly complete entomobirnavirus genome was assembled from adult culicine mosquitoes. The limited number of related entomobirnaviruses discovered thus far has originated from a laboratory cell culture derived from *Drosophila melanogaster*, anopheline mosquitoes from China, culicine mosquitoes from Germany and a C6/36 cell culture of a clinical dengue sample in Brazil. Similar to CYV, CAZV was detected in adult wild-caught culicine mosquitoes but notably, the current study suggests that CAZV also appeared to be present at multiple sites and in both immature and adult culicine mosquitoes.

We identified a partial flavivirus-related sequence in adult chironomids. The sequence was most closely related to Culex flavivirus and other ISFs. Cell fusing agent virus (CFAV) was the first ISF to be discovered over 30 years ago during laboratory studies, in the supernatant medium from a *Stegomyia aegypti* cell line [58]. The first isolations of ISFs from the natural environment include CFAV from Puerto Rico and Kamiti River virus (KRV) from Kenya [4,2]. More recently, *Culex* flavivirus (CxFV) has been isolated and characterised from *Culex* mosquitoes from Asia, the Americas and Africa [59,60,61,62,62,64]. Further ISFs have been isolated from mosquitoes: *Aedes* flavivirus (AeFV) from *Stegomyia* species in Japan [65], Quang Binh virus (QBV) from *Culex tritaeniorhynchus* in Vietnam [66] and Nakiwogo virus (NAKV) from *Mansonia africana nigerrima* in Uganda [63], which are tentative members of the insect-specific group. Importantly, flavivirus RNA has also recently been discovered in phlebotomine sandflies in Algeria [67]. Our study provides the first evidence for ISF sequences from chironomid specimens. Significantly, there has been a recent explosion in the number and diversity of sequences that appear to be related to “insect-specific” flaviviruses amplified from mosquitoes belonging to the culicine genera *Culex*, *Aedimorphus*, *Ochlerotatus* and/or *Stegomyia* that may represent DNA integrations into genomes of mosquitoes [60,61,62,68,69,70,71,72,73,74]. Brackney et al. also recently identified sRNAs related to WNV in apparently uninfected *Culex* mosquitoes [11]. They screened the respective mosquitoes using pan-flavivirus primers and found no evidence for flaviviral infection, concluding that perhaps the *Culex* genome will eventually be found to contain some integrated flaviviral-like sequence in common with *Aedimorphus*, *Ochlerotatus* and/or *Stegomyia* species [42]. The ISF-like sequences detected in adult chironomids in the current study may also represent genome integration(s). Such sequences may confound deep sequencing studies. However, it should be noted that for a subset of samples, the following additional tests were conducted to test for RNA versus DNA amplification: (i) DNase treatment, (ii) absence of RT step and (iii) cell culture and results indicate that flavivirus-like sequences obtained are due to active viral infection.

Regardless of whether the ISF-like sequences detected in the current study originate from virus infection or genome integrations, either would likely result from a long history of contact and interaction between ISF-like viruses and a range of insect taxa. We may hypothesise a common source of virus association or infection for all three dipteran groups (mosquitoes, phlebotomines, chironomids), potentially related to a shared environment and/or transmission and maintenance cycles. Previous studies have presented evidence for vertical transmission of ISFs: CFAV was isolated from both sexes of a number of mosquito species [4] and the presence of CxFV RNA has been detected in the eggs and larvae of *Culex pipiens* from a laboratory colony [74]. Chironomid larvae feed on organic matter in aquatic sediment [75] and share habitats with both mosquito and phlebotomine larvae, which are, respectively, filter-feeders that feed on microorganisms and organic debris and scavengers that feed on microorganisms associated with decaying vegetation, manure and other nutrient-rich material. Crucially, chironomid adults are non-biting, utilising food sources that include nectar and honeydew. Like many mosquito species, phlebotomine females are blood feeders. Taken together with the current study, we suggest that available data indicate a high potential for both vertical and horizontal transmission of ISF-like viruses across a range of dipteran species, which may involve any or all of the following: (i) trans-ovarial transmission, (ii) potential infection at the larval stage, possibly linked to a shared aquatic habitat, (iii) potential infection via shared sugar-rich food sources such as plant exudates or even (iv) transmission via shared microparasites. Therefore, appropriate future study includes the field testing of dipteran eggs versus larval and adult specimens with complementary experimental infection studies. To our knowledge, the potential involvement of shared microparasites, e.g. mites, in ISF and other related viral transmission cycles has never been addressed. Protocols used in the current study produced sequencing templates comprising exclusively RNA. However, some previous studies of ISFs have not included a number of controls to more easily distinguish, in samples such as adult dipteran specimens, genome integrations from *bona fide* viruses. Such controls must be incorporated in future studies, namely testing via (i) lack of reverse transcriptase and (ii) DNase-treatment of samples.

We also detected a sequence related to the large segment of members of the family *Bunyaviridae* in adult culicine mosquitoes. The contig contained a single long ORF that showed greatest similarity to members of the genus *Phlebovirus*, and the currently unclassified GOUV which was originally detected in specimens of the mosquito genera *Anopheles* (anophelines), *Culex* and *Uranotaenia* (culicines) in West Africa. Here, we provide evidence for a related novel virus from mosquitoes collected in France. Further field studies are necessary to allow the potential full isolation of this strain, which considering its divergence from GOUV, may constitute a novel genus within the family *Bunyaviridae*. In addition, we detected sequences in adult anopheline mosquitoes and adult culicine mosquitoes with similarity to the orbivirus VP1/RdRp-encoding segment (segment 1). Orbiviruses infect a range of hosts, including cattle, goats and sheep. The three economically most important orbiviruses are bluetongue virus, African horse sickness virus and epizootic hemorrhagic disease virus, all of which are transmitted by *Culicoides* species (midges of the dipteran family Ceratopogonidae). The detection of novel orbivirus sequences in both culicine and anopheline mosquitoes may warrant further study, with the aim of viral isolation and full characterisation. From adult chironomids and adult culicine mosquitoes, we also identified sequences related to *Spissistilus festinus* virus 1 (SpFV1) and *Circulifer tenellus* virus 1 (CiTV1). SpFV1 and CiTV1 were originally isolated from hemipteran insects (order Hemiptera, plant-feeding true bugs with sucking mouthparts) whereas the current study provides evidence for related sequences present in dipteran insects (both chironomids and culicine mosquitoes). 

We see parallels among many of the viral families encountered in this study, particularly the entomobirnaviruses and flaviviruses, which include recently discovered potential strains of highly divergent viruses in a range of species, e.g. ESV, CYV and MXV (entomobirnaviruses) and *Culex* flavivirus CxFV, Nakiwogo virus NAKV and Kamiti River virus KRV (flaviviruses), related to isolates first detected in the laboratory, e.g. DXV and CFAV. First, these novel naturally occurring strains provide useful comparisons for research on cell culture adapted strains such as DXV, and tools to examine the response of multi-passaged insect cell lines such as C6/36. In the wider picture, it is becoming clear in an increasingly large number of viral families that there is a diverse and highly prevalent background of transmission and maintenance of numerous viral strains across a range of host taxa against which known pathogens are evolving. The relevance of this genetic diversity in terms of potential competitive exclusion, superinfection or potential recombination events requires extensive study.

Although the sequences identified in the present study are certainly of viral origin, our methods do not allow us to definitively distinguish sequences of *bona fide* viruses from RNA transcripts of genome-integrated virus sequences. In the cases of the CBPV-like virus AACV and the entomobirnavirus CAZV, the presence of both segments, the high coverage, the presence of multiple well-supported (and often synonymous) polymorphisms and the presence of terminal motifs strongly suggest that these sequences derive from *bona fide* replicating viruses. The origin of the other sequences identified is less certain. Further investigations aimed at the isolation and full characterisation of viruses from a wide range of insects is a priority. The narnavirus-, mitovirus- and partitivirus-like sequences obtained in the current study may derive from fungal (or other) contaminants of the insect preparations, either external or internal (e.g. insect gut contents). Similarly, a number of plant-virus-like sequences (not reported) were detected in samples 7 and 9. During the analysis, we found a number of other virus-related sequences (not reported) that appeared to have fragmented ORFs (internal stop codons or frameshifts in unexpected positions, or chimeric sequences), and some of these likely represent transcribed genome-integrated sequences. A large number of rhabdovirus-like sequence fragments were identified but were not further analysed as genome integration of rhabdovirus-like sequences has been shown to be particularly common in arthropod genomes [43,45]. 

In some cases, genome-integrated sequences appear to provide a functional benefit to the host, and are retained (exaptation) and may be subjected to purifying selection [45,76,77]. Thus, an excess of synonymous SNPs over nonsynonymous SNPs is not necessarily a sign of a *bona fide* (non-integrated) virus sequence, e.g. if the host species sample preparation is not genetically uniform or if related sequences have been integrated at multiple sites within the same genome. Once integrated, such sequences evolve at a rate many orders of magnitude more slowly than replicating RNA viruses, and thus such sequences may provide a window on the time-scale of evolution of RNA viruses, which appears to occur at a much slower rate than what one would infer from extrapolating short-term evolutionary rates [77].

Most of the sequences we identified contained the viral RdRp. This may be because the RdRp tends to evolve more slowly than other regions of an RNA virus genome; hence, it is easier to identify the RdRp in highly divergent viruses based on blastp analysis. This is also true for integrated sequences: whereas the RdRp of ancient RNA viruses, e.g. 10-100 mya, may resemble the RdRp of extant RNA viruses, other proteins of integrated ancient RNA viruses may not be detectable by blastp comparison to extant viruses. It is possible that applying structure-based homology search tools, such as hhpred [31], to our complete set of contigs might enable detection of other virus genes (e.g. jelly-roll-fold structural proteins); however such an analysis is not easily accomplished due to CPU constraints.

In this study, we report the discovery of viruses in wild-caught dipteran insects using deep sequencing of RNA. In our approach, total small RNAs were isolated from a field collection, sequenced via Illumina and assembled into contigs via Velvet and analysed using a custom-built bioinformatics workflow process. Results were used to inform total RNA sequencing of samples of particular interest (in this case, samples from three different ecological sites). This strategy enabled the construction of two complete or nearly complete virus genomes and numerous other novel virus sequences with minimal *a priori* knowledge. Except for the entomorbirnavirus-related sequences, all exhibited low amino acid sequence identities (24-42%) to known viruses.
